# Supplementary material for: Multiomic profiling of medulloblastoma reveals subtype-specific targetable alterations at the proteome and N-glycan level
Source: Nat Commun. 2024 Jul 24;15:6237. doi: 10.1038/s41467-024-50554-z (PMC11266559; doi:10.1038/s41467-024-50554-z)
Supplement: Supplementary file 1 — Supplementary Information [file 41467_2024_50554_MOESM1_ESM.pdf]

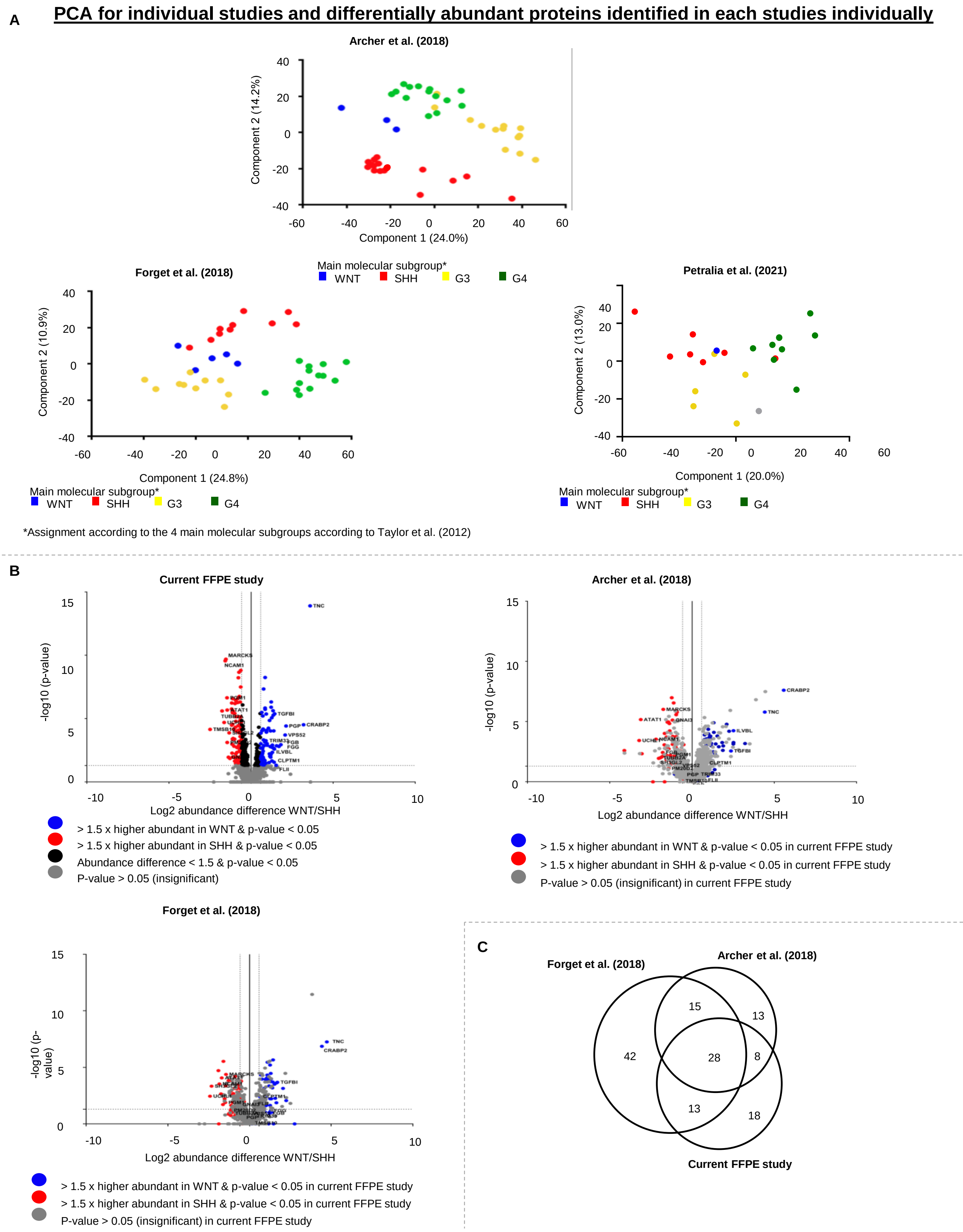

Supplementary Figure 1: A Scatter Plot visualization of the first 2 principal components in NIPALS PCA, based all proteins, quantified in individual cohorts (Archer et al (2018):14522 proteins, n = 45, Forget et al.(2018): 3890 proteins, n =38 , Petralia et al (2021): 5680 proteins, n=22. Samples were colored according to the main molecular subtype (only samples with successful proteome subtype assignment were used for PCA. **B** Volcano Plot visualization of two sided, two-sided unpaired, t-testing results, showing the abundance distribution of differential abundant proteins between SHH and WNT type MBs in the current FFPE study and the fresh frozen tissue datasets, provided by Archer et al. (2018) and Forget et al. (2018). For T-testing proteins were reduced to 2968 proteins, identified in the current FFPE study (n=62). Proteins with a p-value < 0.05, that showed a foldchange > 1.5 between tested groups were considered statistically significant and differential abundant. **C** Venn diagram, comparing statistically significant differential abundant proteins between SHH and WNT type MBs between three different proteome studies on human MB tissue (Forget et al. (2018); Archer et al. (2018); current FFPE study. To enable comparability across studies proteins were reduced to 1594 proteins, found at least 2 times in all studies. . n represents biologically independent human samples.

Correlation between matched FF and FFPE samples

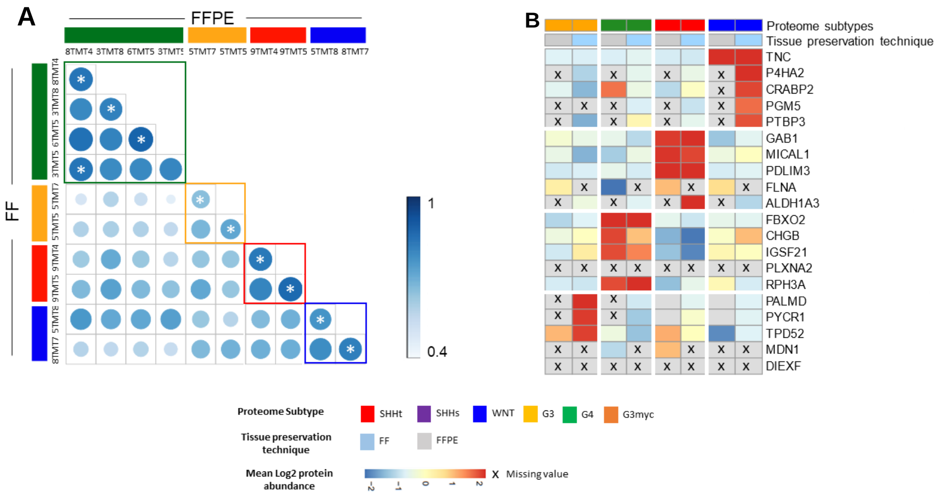

Supplementary Figure 2: A Corrplot showing pearson correlation between FF sample (n = 10) s on Y-axis and their respective FFPE samples (n = 10) on the X-axis annotated for proteome subtype. Asterisks mark highest Pearson score. **B** Mean of biomarkers for each proteome subtype (defined in the study). n represents biologically independent human samples.

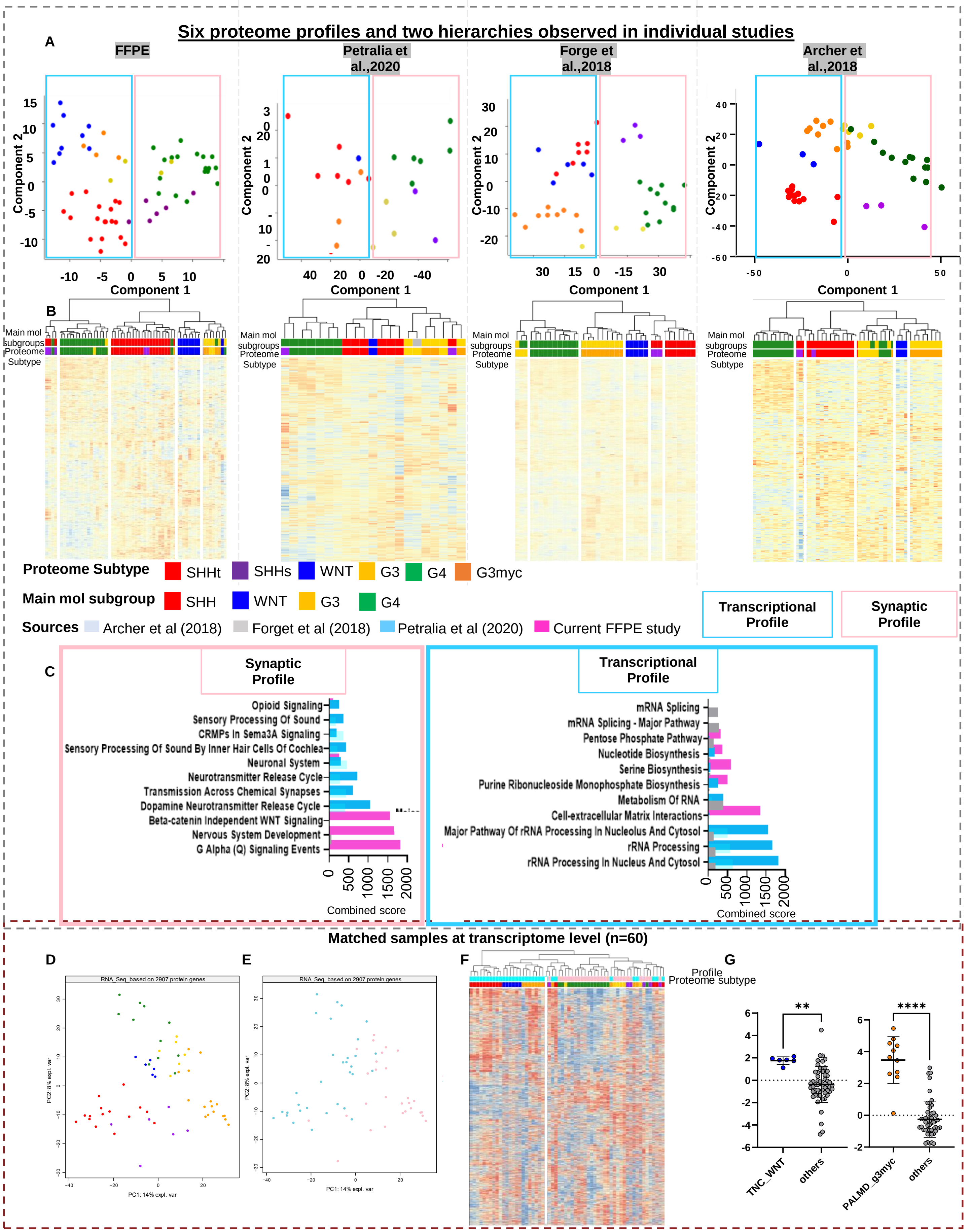

Supplementary Figure 3: **A** PCA on each individual dataset (Petralia et al,2020, n = 22, Forget et al,2018, n = 38, Archer et al,2018, n =45) reflecting two main profiles – synaptic (pG3, pG4 and pSHHs) and transcriptional (pG3myc, pWNT, pSHHt) **B** Hierarchical clustering using 100% valid values for each individual dataset (Pearson correlation and ward.D2 linkage) **C** Enriched pathways in each individual datasets – two-sided, unpaired t-test based on two profiles synaptic and transcriptional profile where serine and nucleotide biosynthesis support higher transcription and translation **D** PCA showing separation of six proteome subtypes at RNAseq level for matched samples (n = 60,using 3990 genes corresponding to the proteins quantified in the main cohort, n=60, integrating FFPE RNA seq and Archer et al,2018, batch corrected using harmonizR)) and **E** based on two main profiles (synaptic and transcriptional) **F** Hierarchical clustering using 3990 genes using Pearson correlation and ward.D2 linkage-separation based on two main profiles and reflecting the six proteome subtypes (transcriptional profile = pWNt, pSHHt,pG3myc, synaptic = pSHHs,pG3 and pG4) **G** Biomarkers identified at proteome level for pWNT (TNC, n_{WNT} = 6, n_{others} =54, pvalue = 0.02) and pG3myc (PALMD, n_{G3myc} = 11, n_{others} =49, pvalue < 0.001, unpaired t-test), data presented as +/- SD. n represents biologically independent human samples.

Protein abundances for housekeeping proteins and number of proteins quantified based on year

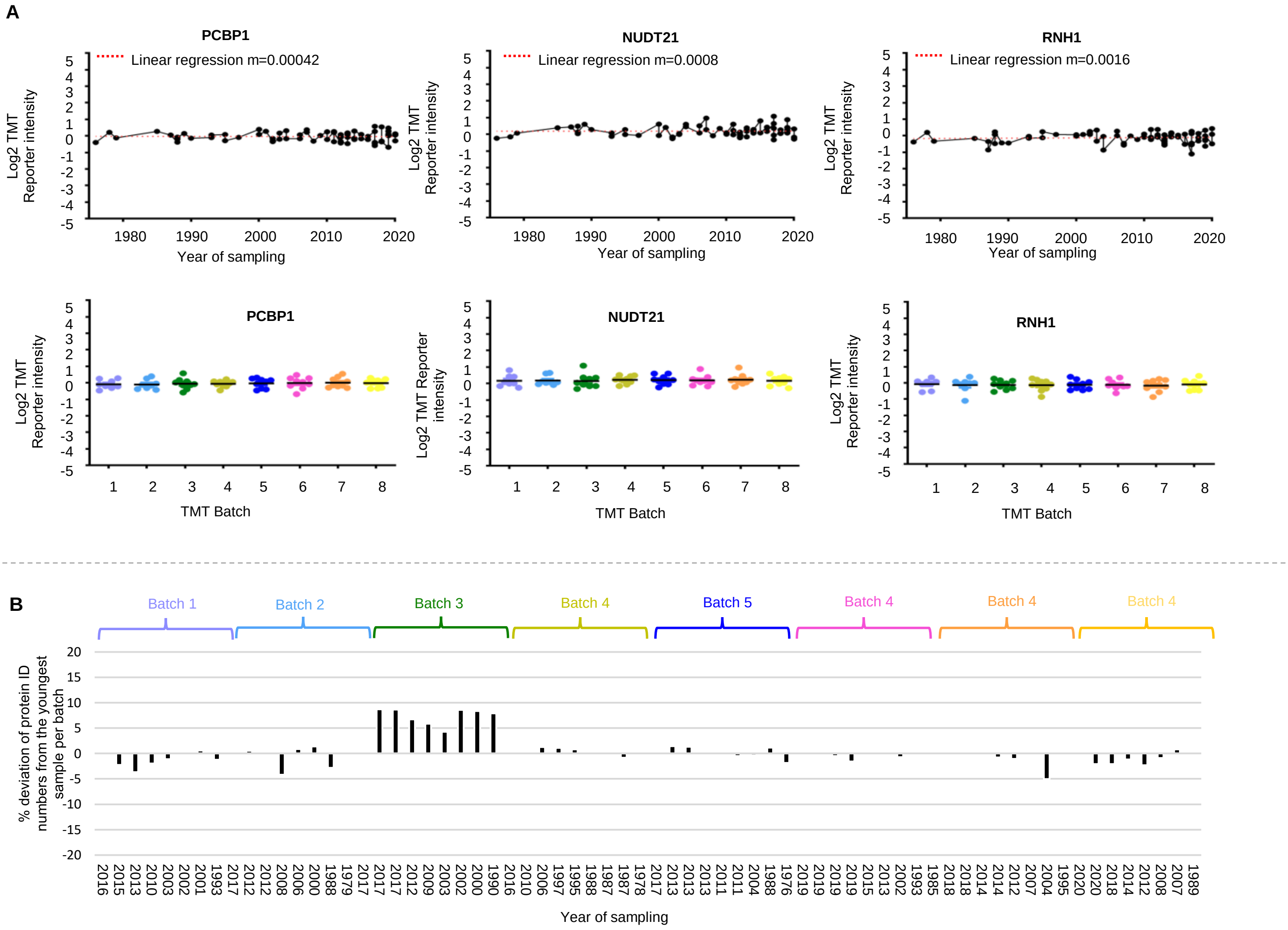

Supplementary Figure 4: A Log2 TMT Reporter intensity for selected housekeeping proteins per measured TMT Batch and across sampling years. Normalized, TMT Batch-Effect corrected protein abundances were used. Each dot represents one independent biological sample. **B** Percentage deviation of the total number of identified proteins from the identified proteins of the youngest sample per batch.

A

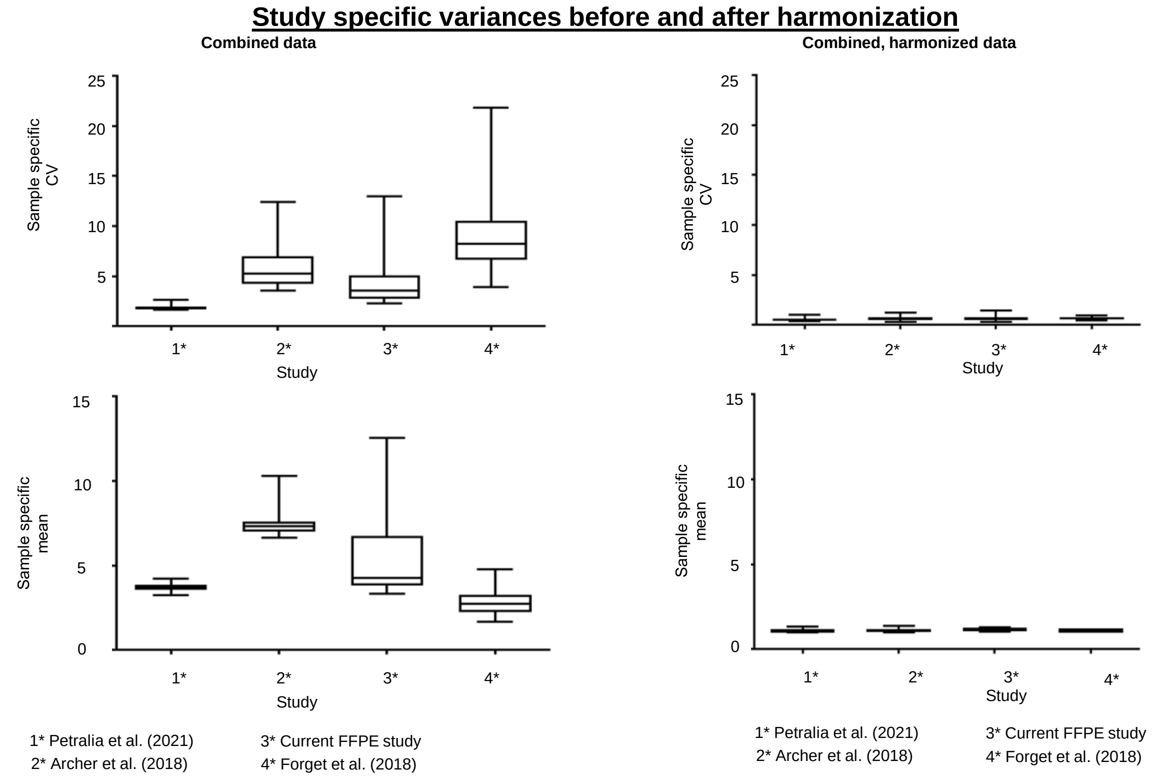

B

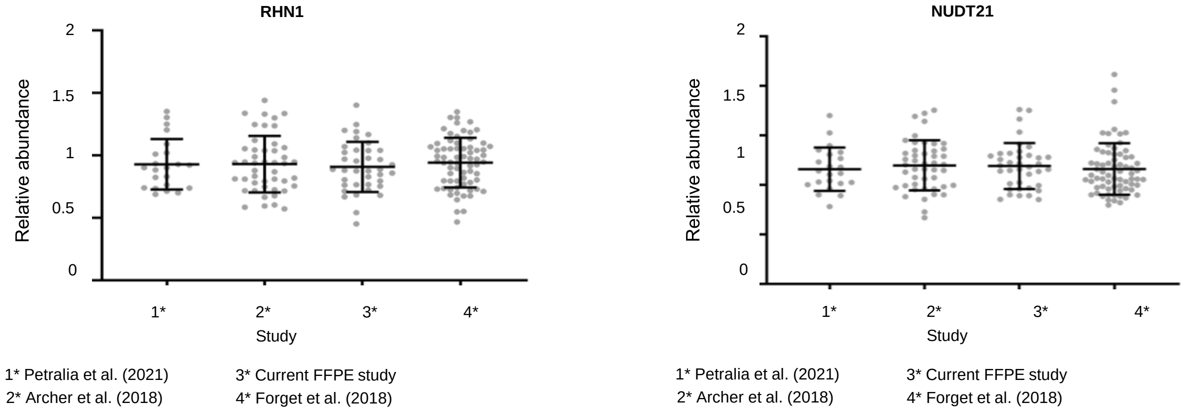

Supplementary Figure 5: A Sample specific CV and mean for each batch prior to and after the usage of ComBat (L/S scaling, parametric Bayesian framework) in the HarmonizR framework prior to and after data harmonization across studies. In boxplots, 50 % of the data points are inside the box (Q1 (Quartile 1) being the lower bound of the box (25 %), Q3 being the upper bound of the box (75 %)). Whiskers show all values beyond the box without outliers. Outliners were defined as Q3 + 1.5 * IQR (Interquartile range) (upper outlier) and Q1-1.5 * IQR (lower outlier). IQR being Q1-Q3. **B** Protein abundance distribution of housekeeping proteins NUDT21 and RHN1 across different studies after data harmonization, data presented as +/- SD.

Classification of samples based on Random forest classifier

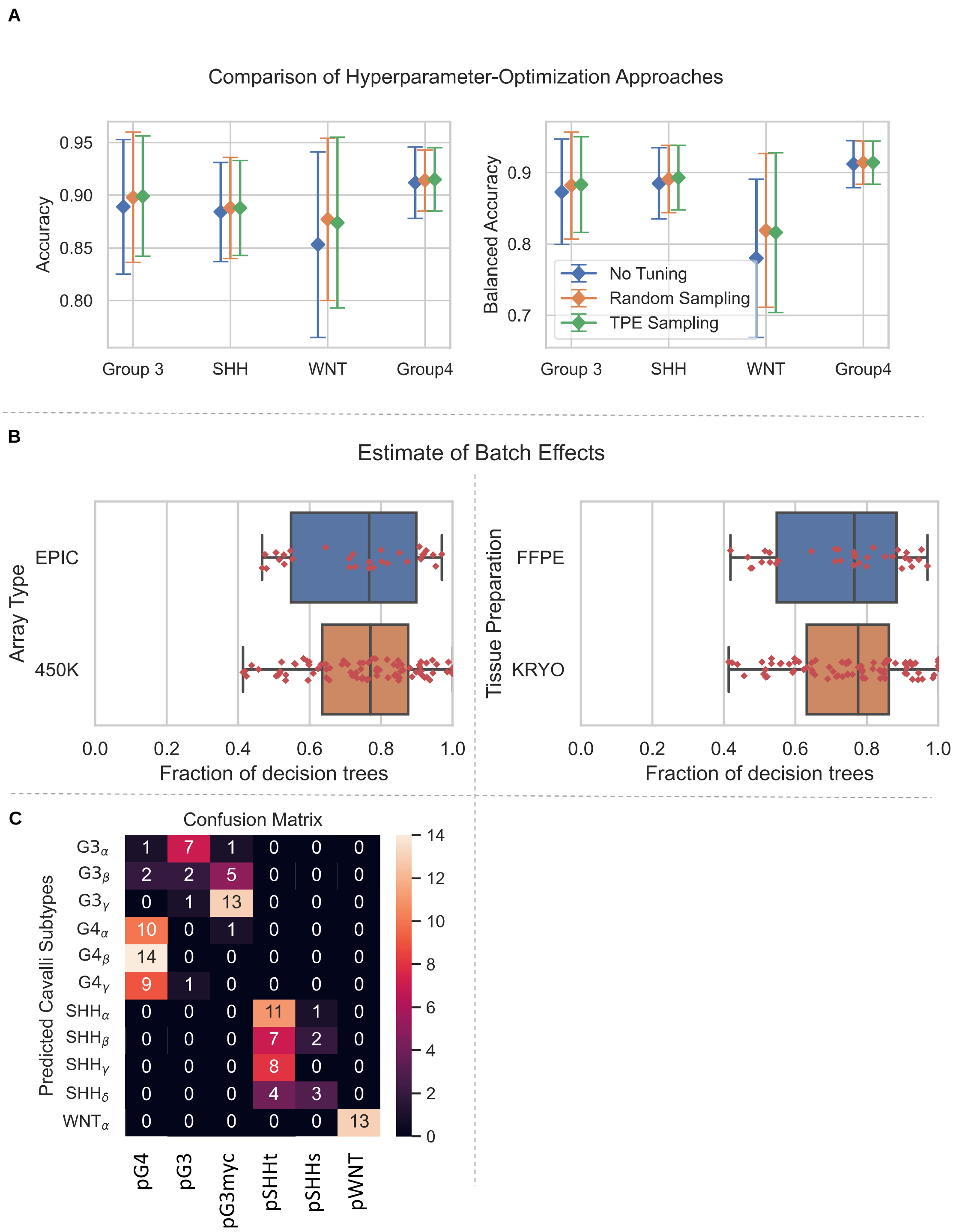

Supplementary Figure 6: A Comparison of different approaches to hyperparameter-optimization for the RandomForest-algorithm in order to classify samples into subgroups based on DNA-methylation as described by Cavalli et al (2017), data presented as man +/- SD. In general, TPE-Sampling yields the best accuracy (left) and balanced accuracy (right) on randomly drawn validation sets. **B** Estimation of the impact of array type and type of tissue preparation on the classification. The number of DecisionTrees voting for the predicted class shows no significant relationship with both processing parameters (Kruskal-Wallis-H-test p-value 0.511 for the array type and 0.540 for the type of tissue preparation), dots represent single value, box represents Median with Q1 to Q3, error bars show Q1-1.5*IQR and Q3+1.5*IQR. IQR=inter-quartile range . **C** Confusion matrix for the predicted Cavalli-subtype and the proteomic subtypes from this study. n represents biologically independent human samples.

Drugs associated with SNARE complex and EIF2 signalling

A SNARE Complex

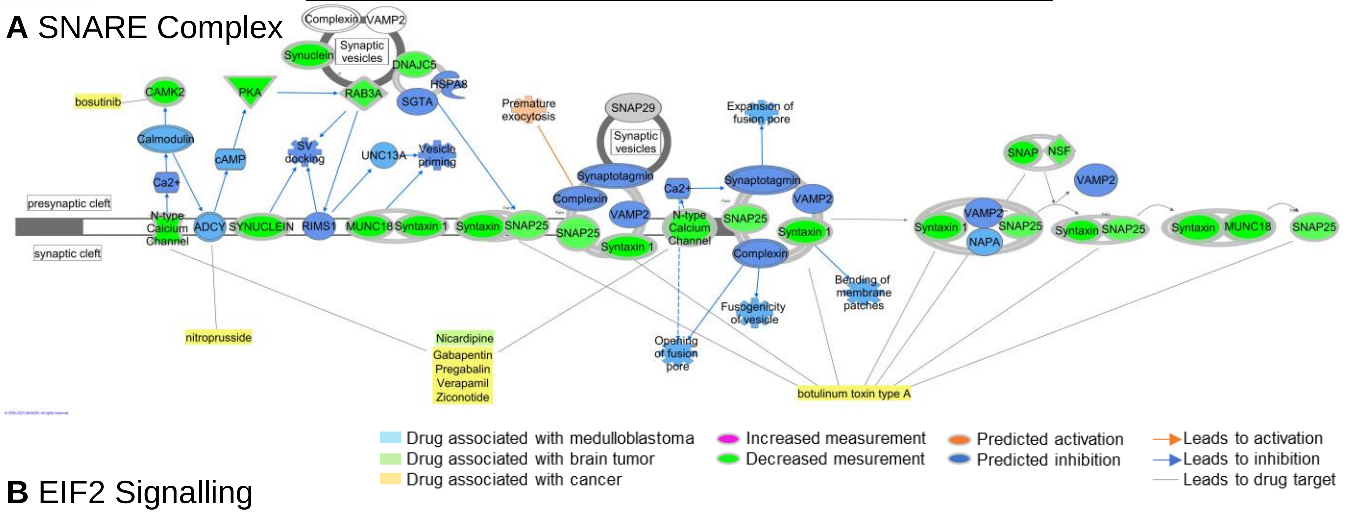

B EIF2 Signalling

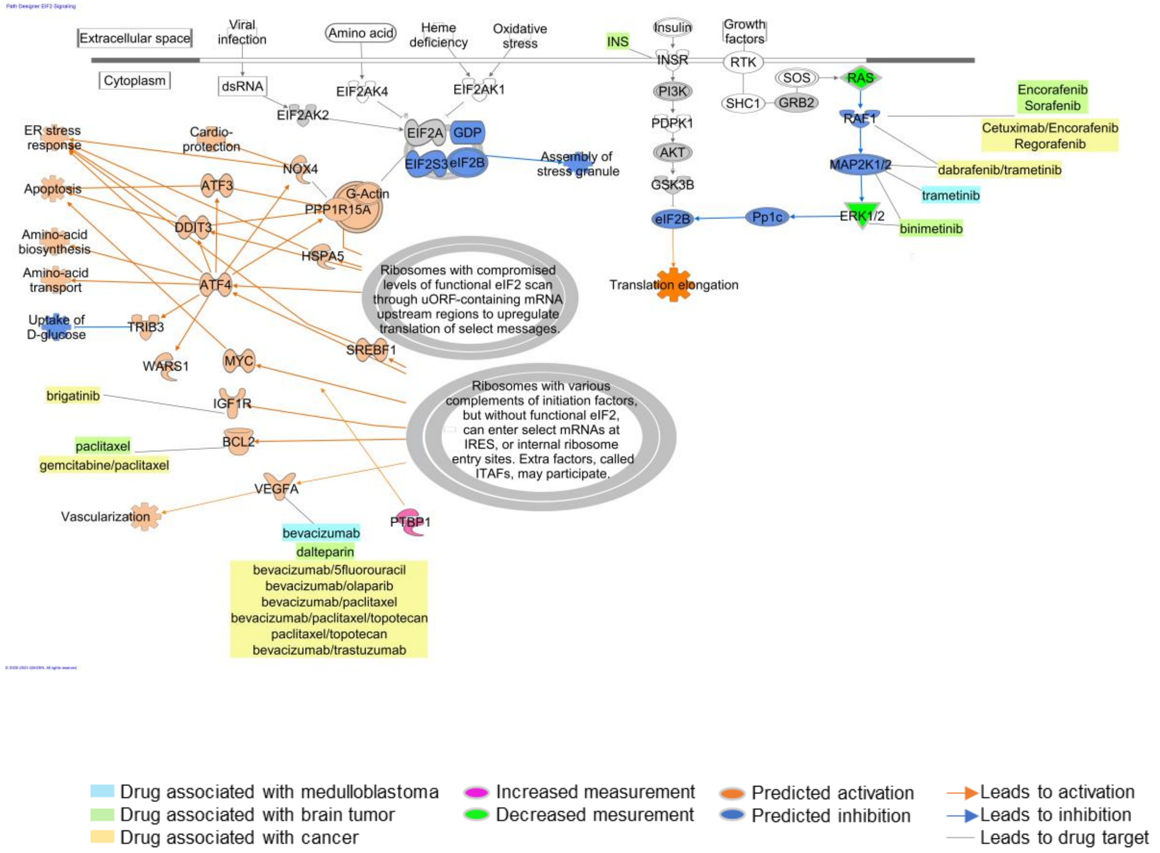

Supplementary Figure 7: **A** Drugs associated with SNARE complex and **B** EIF2 signalling found using Ingenuity pathway analysis.

Consensus clustering at DNA methylome level

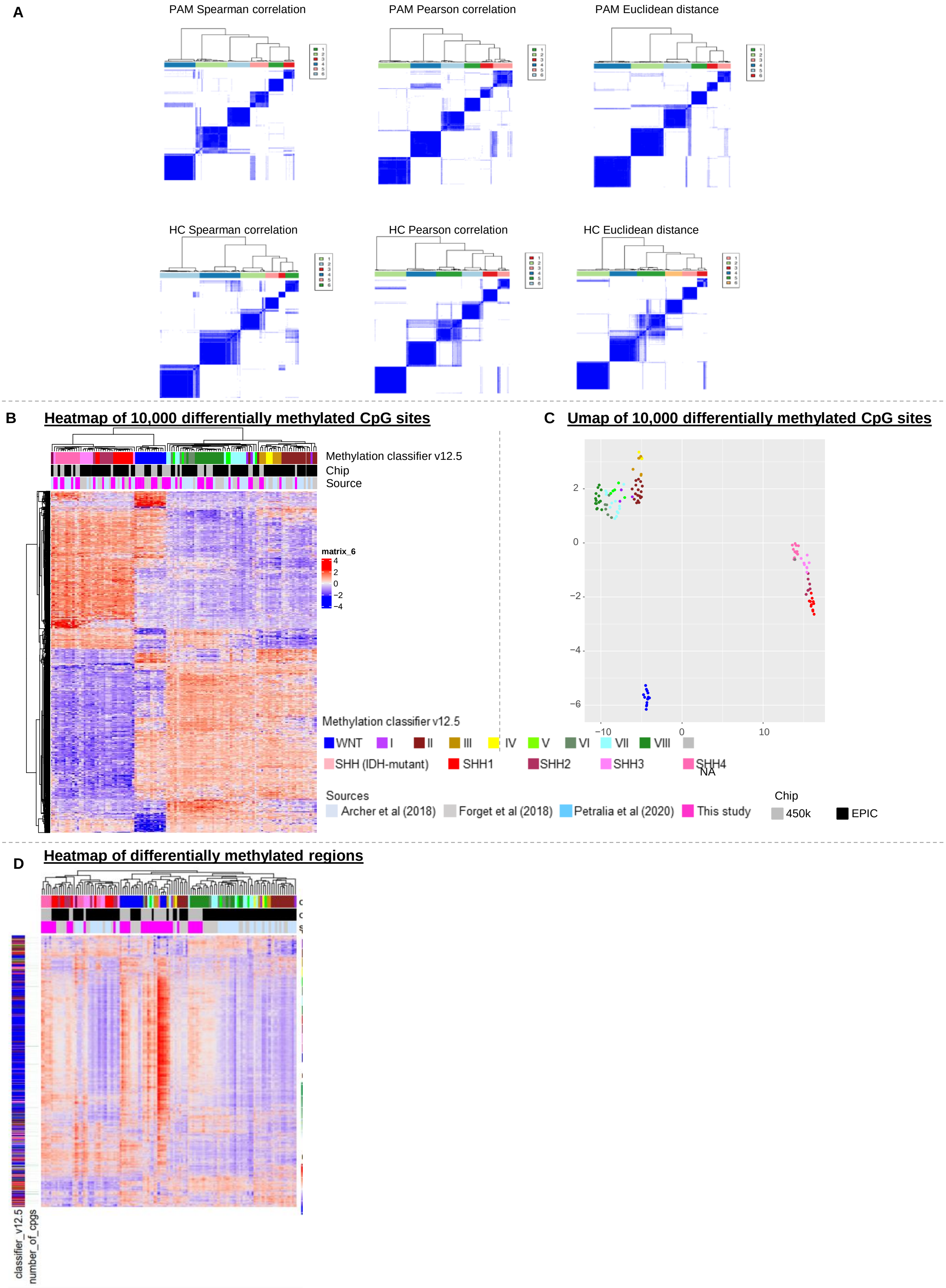

Supplementary Figure 8: A The optimal clustering methylome data, as determined using Pearson correlation, Spearman correlation and Euclidean distance as distance metric for hierarchical clustering (HC) and k-meloids clustering (PAM) respectivley. *k*, number of clusters. Consensus scores are indicated using a color scale from white (samples never cluster together) to blue (samples always cluster together). **B** Heatmap of 10,000 most differentially methylated CpG sites for n = 117 samples (M-values) hierarchically clustered using pearson correlation and ward.D linkage (moderated t-statistic corrected for multiple testing using Bonferroni-Hochberg correction and p-value < 0.05).C. Umap based on these differentially methylated CpG sites **D** Hierarchichal clustering of 10,000 differentially methylated DMRs, clustering based on the 14-subtypes using MNP v12.5 RandomForest Classifier. n represents biologically independent human samples.

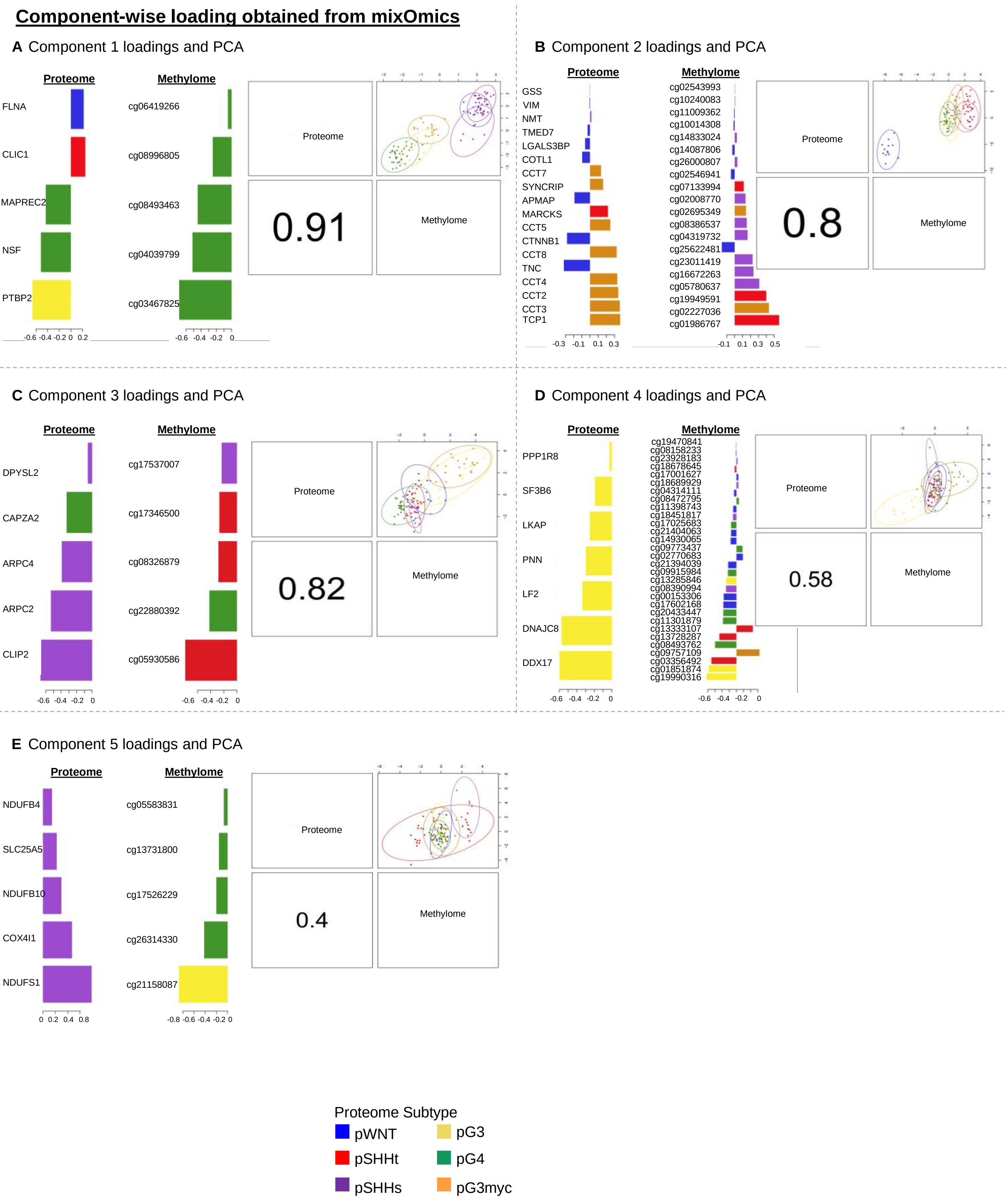

Supplementary Figure 9: A-E Integration of DNA methylome (n = 115 samples,10,000 differentially methylated CpG sites) and proteome data (115 samples, 3990 proteins) was achieved using sparse partial least squares discriminant analysis (sPLS-DA). This integration reduced the data into six components (artificial variables made up of weights of original variables contributing to the respective component). **A-E** The barplots represent the loading value (defined as the weight assigned to each feature to determine their contribution to the selected component) and the colors represent the proteome subtype to which the feature contributes. The length of the bar plot represents the absolute loading value and the direction depicts whether the loading value is positive or negative. The corresponding PCA shows how well the selected features for each component separate the six proteome subtypes. . n represents biologically independent human samples.

MixOmcs using DMRs instead of CpGs

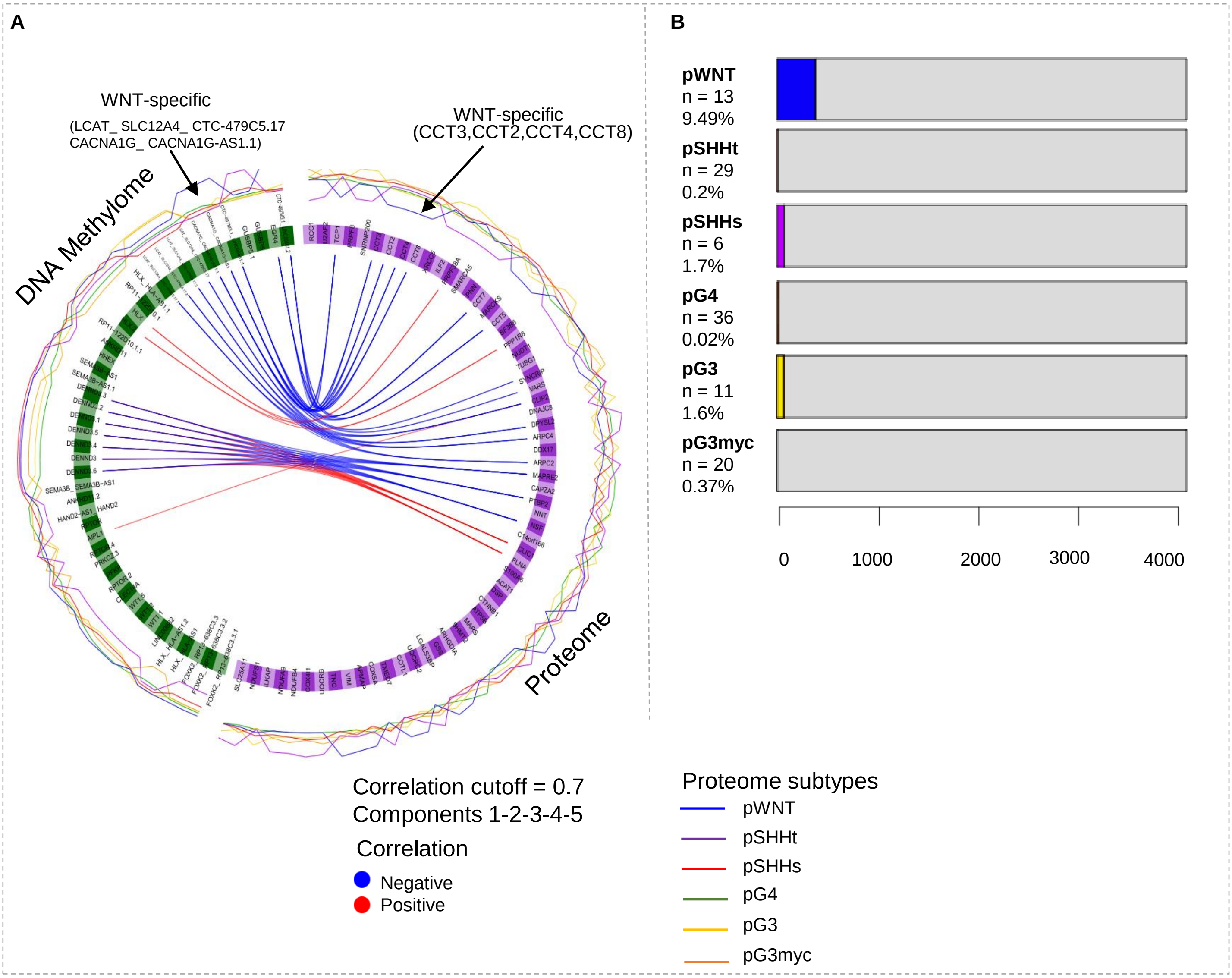

Supplementary Figure 10: A Circos plot using 10,000 differentially methylated regions integrated with 3990 proteins from the main cohort (n = 117 samples). Plotted here are features selected on 5 components showing a correction of > 0.7.

B. Barplot representing the number of proteins correlating with the differentially methylated region belonging to their own gene. (pearson correlation > 0.7 are plotted). n represents biologically independent human samples.

Differences in pSHHt and pSHHs at transcriptome level and using Ki67 stainings

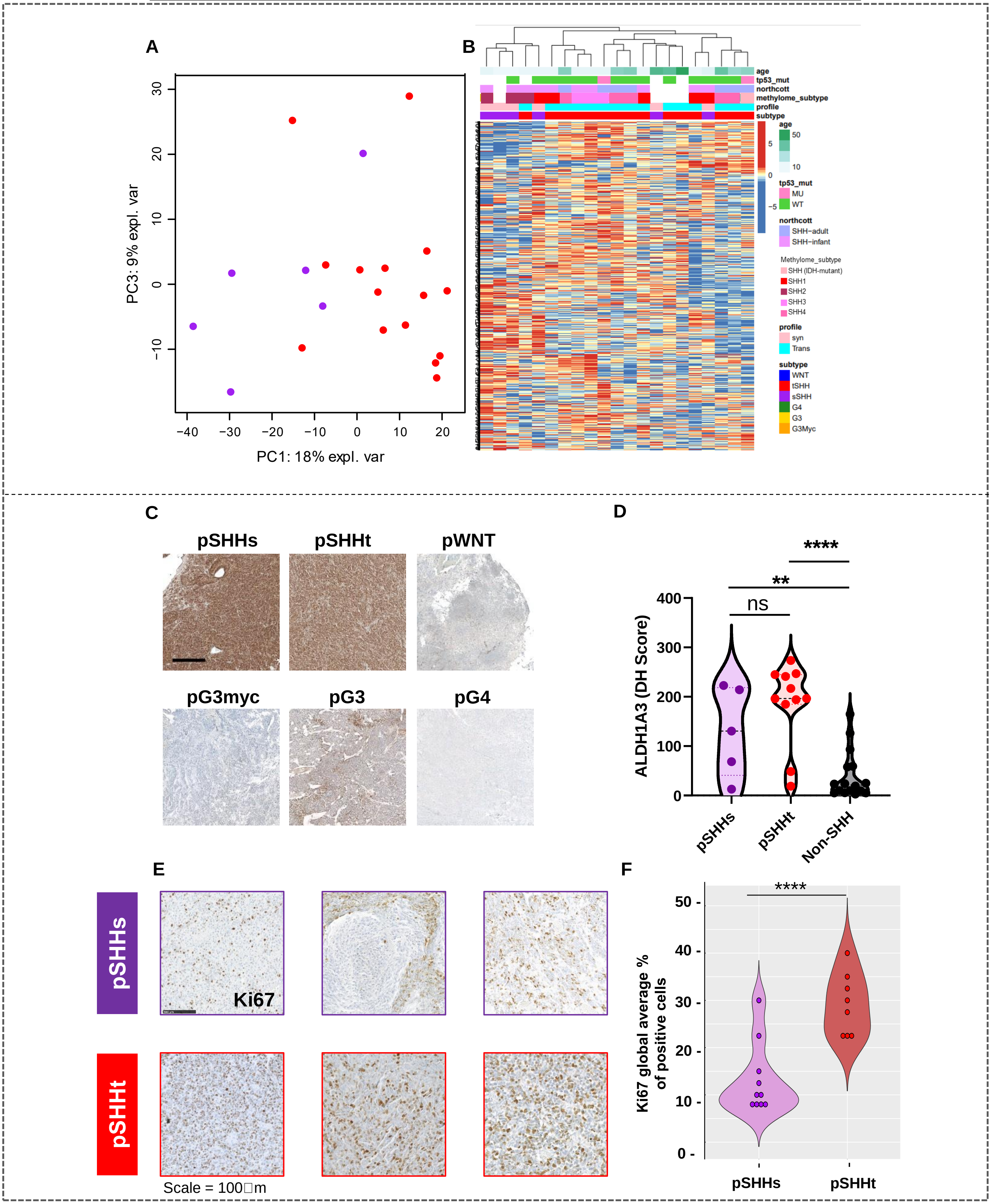

Supplementray Figure 11: A PCA and **B** Hierarchical clustering using 100% valid values, Pearson correlation and ward.D2 linkage only in SHH samples (n=21) **C** Representative images of immunohistochemical stainings against ALDH1A3. A strong cytoplasmic signal was detected in pSHHt and pSHHs MBs. **D** Digital quantification of ALDH1A3 DAB signals showed a significantly enhanced digital histoscore (DH Score) for pSHHt and pSHHs compared to all other MB subtypes (n_{pSHHs}=5, n_{pSHHt}=11, $n_{non\text{-}SHH}$=16, $p_{pSHHt\_vs\_pSHHs}$=0.0224, $p_{pSHHs\_vs\_non\text{-}SHH}$=0.009, $p_{pSHHt\_vs\_non\text{-}SHH}$=<0.0001, unpaired t-test). Scale bar is 500 µm.
E Representative images for Ki67 stainings. Higher number of cells stained positive for Ki67 in pSHHt compared to pSHHs. **F** pSHHt subtypes show significantly higher percentage of Ki67 positive cells compared to pSHHs group (n_{pSHHs}=6, n_{pSHHt}=6, $p_{pSHHt\_vs\_pSHHs}$=<0.0001, unpaired t-test. . n represents biologically independent human samples.

Amino acid and metabolite distribution

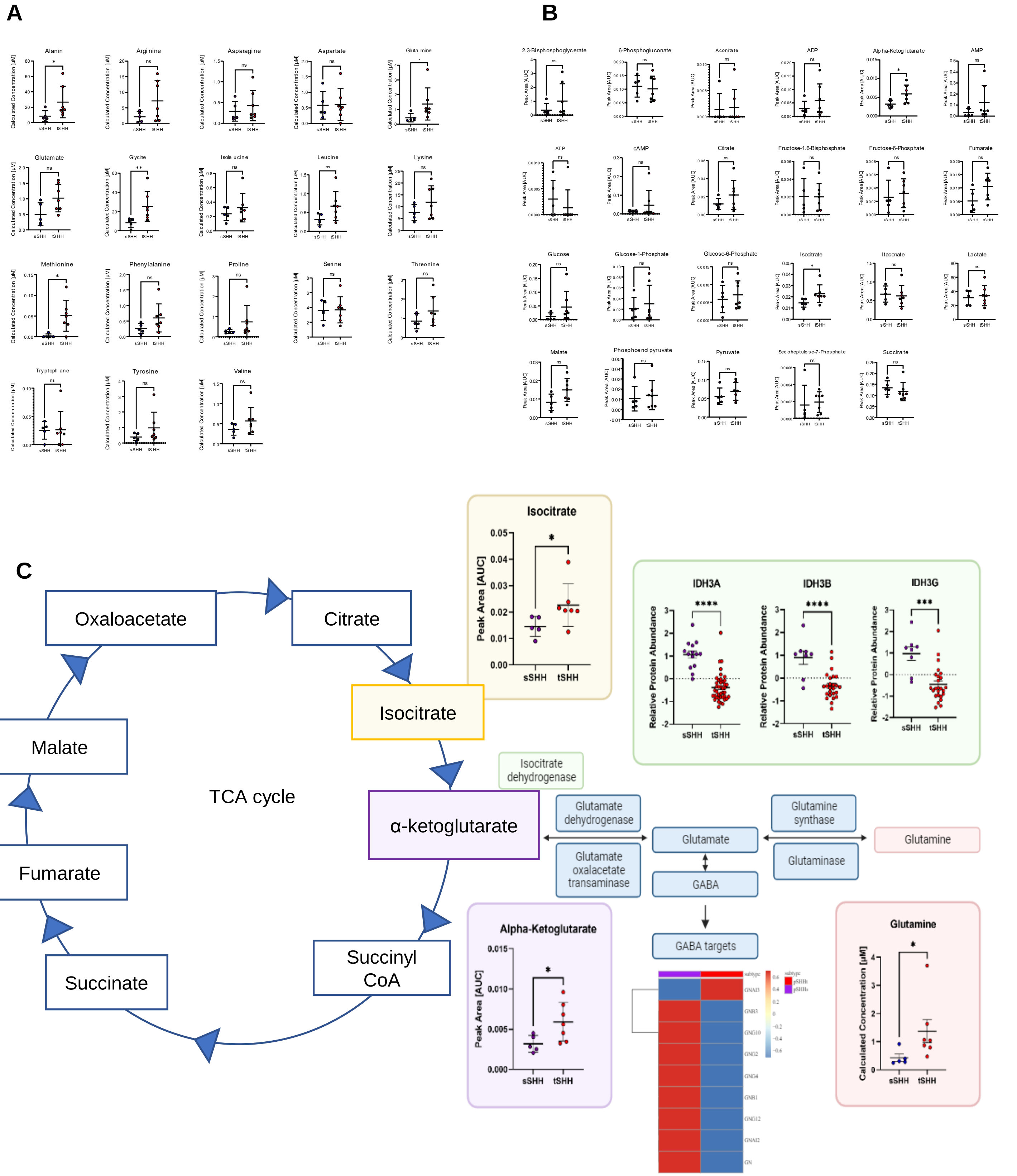

Supplementary Figure 12: : A Amino acids and **B** metabolite distribution between the two SHH proteome subtypes, normalized to internal standards and B metabolite distribution **C** Significantly different amino acids and metabolites (Mann-Whitney T-test p-value < 0.05) showing higher abundance in the pSHHt group compared to pSHHs group plotted along with significantly different proteins (two-tailed, unpaired t-testlog2FC > 1.5 and p-value < 0.05) with high abundance in pSHHt. Lower abundance of Isocitrate and alpha ketoglutarate with high abundance of isocitrate dehydrogenase proteins points at higher consumption of these metabolites in the pSHHs group and hence high abundance of GABA targets which are involved in synaptic signaling

<u>**Immunhistochemical stainings for cMYC**</u>

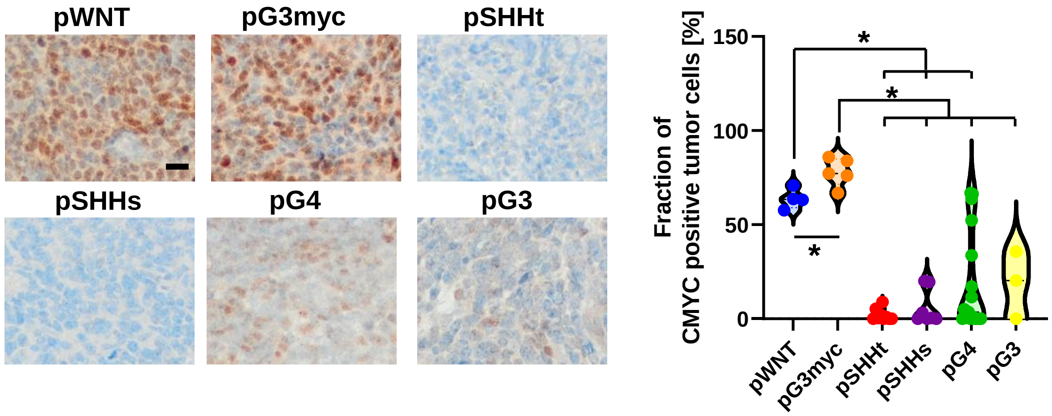

Supplementary Figure 13A:

Representative images of Immunohistochemical stainings against CMYC with quantification of the fraction of CMYC positive tumor cells. A strong nuclear signal was detected in a majority of tumor cells in pWNT (n=4) and G3myc (n=5) MBs ($Mean_{pWNT}$: 63.9 %, $Mean_{pG3Myc}$ = 78.0 %), whereas all other MB subtypes showed lower fractions ($mean_{pSHHs}$ =6.2 %, $mean_{pSHHt}$ = 2.0 %, $mean_{pG3}$ =18.7 %, $mean_{pG4}$ = 18.9 %). *: $p_{pG3myc\_vs\_pWNT}$=0.03, $p_{pG3\_vs\_pWNT}$=NS, $p_{pG4\_vs\_pWNT}$=0.02, $p_{pWNT\_vs\_pSHHt}$=0.002, $p_{pWNT\_vs\_pSHHs}$=0.006, $p_{pG3myc\_vs\_pSHHt}$=0.001, $p_{pG3myc\_vs\_pSHHs}$=0.002, $p_{pG3myc\_vs\_pG3}$=0.03, $p_{pG3myc\_vs\_pG4}$ <0.0001,, unpaired Mann-Whitney-test). Scale bar is 10 µm . . n represents biologically independent human samples.

PALMD and TNC methylome data

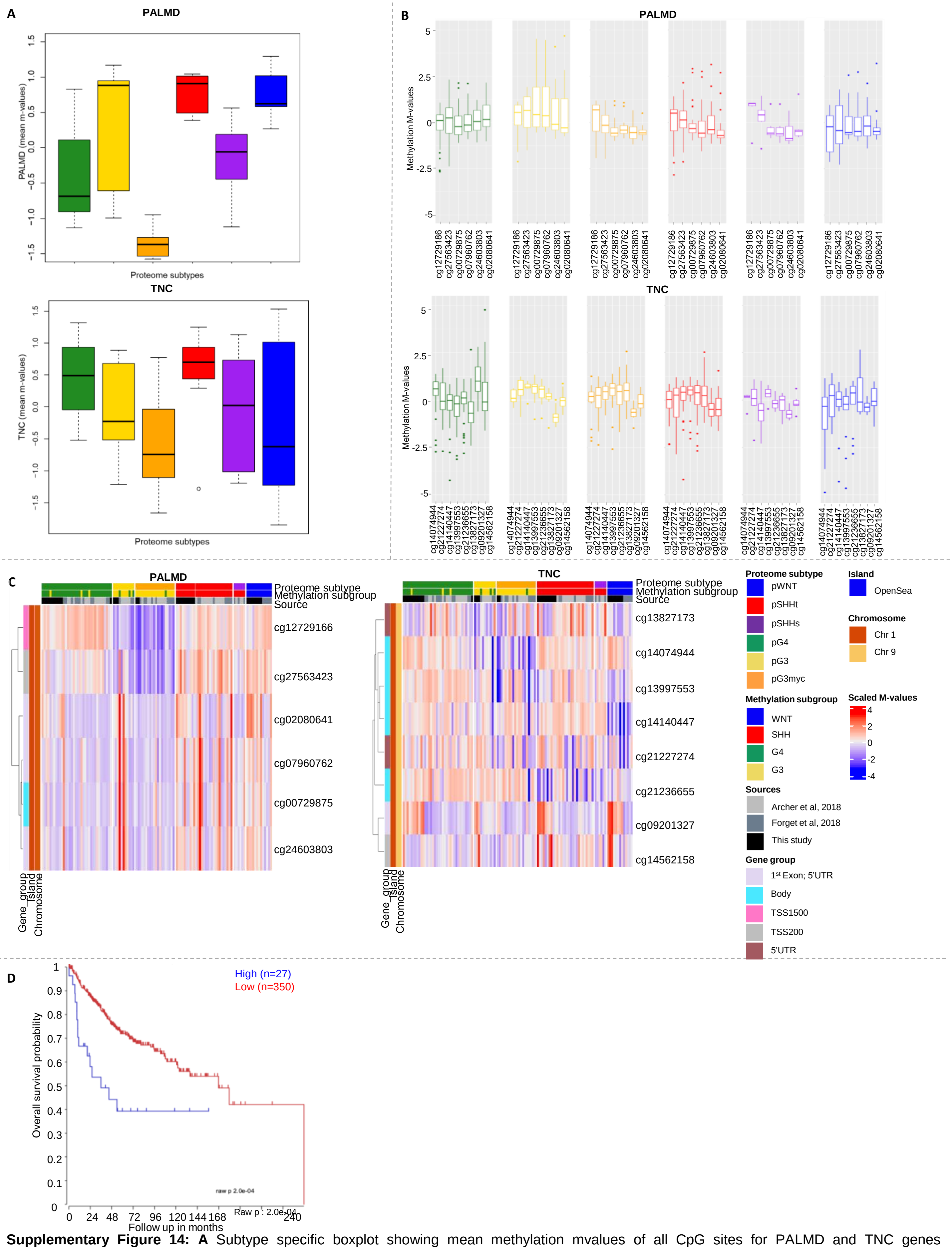

Supplementary Figure 14: A Subtype specific boxplot showing mean methylation mvalues of all CpG sites for PALMD and TNC genes respectively across proteome subtypes (boxplots represents Median with Q1 to Q3, error bars show Q1-1.5*IQR and Q3+1.5*IQR. IQR=inter-quartile range), for each subtype. **B** Boxplots displaying **m**ethylation mvalue of all individual CpG sites of PALMD and TNC in six proteome MB subtypes (n=117) (boxplots represents Median with Q1 to Q3, error bars show Q1-1.5*IQR and Q3+1.5*IQR. IQR=inter-quartile range), . **C** Heatmap displaying the methylation of individual CpG sites of PALMD and TNC along with CpG site information: gene group, island and chromosome. **D** Survival plot showing differences in survival based on PALMD gene expression from the Cavalli dataset (Cavalli et al,2018, n_{high} = 27, n_{low} = 350). The plot was generated with the 'R2: Genomics Analysis and Visualization Platform (http://r2.amc.nl)). n represents biologically independent human samples.

CCT- old cohort and cavalli

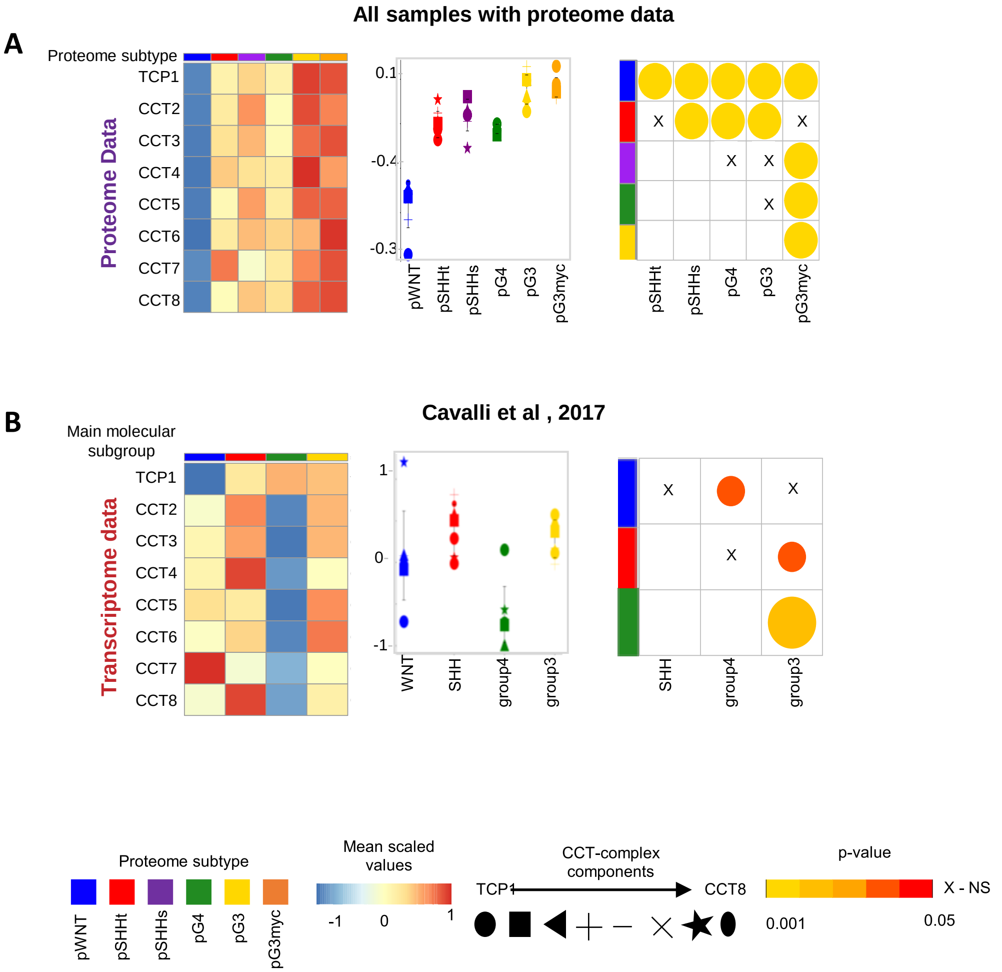

Supplementary Figure 15: A Left : Heatmap displaying CCT-complex components abundance across the six- proteome subtypes (mean per subtype, n=167) Middle : quantification as dotplots for mean per CCT- component per, data are presented as +/- SD and Right : corrplot displaying the pvalues of the t-test results for each comparison in whole proteome data subtype ($p_{pWNTvspSHHt}$ < 0.0001, $p_{pWNTvspSHHs}$ < 0.0001, $p_{pWNTvspG3}$ < 0.0001, $p_{pWNTvspG3myc}$ < 0.0001, $p_{pWNTvspG4}$ < 0.0001, $p_{pSHHtvspSHHs}$ < 0.001, $p_{pSHHttvspG3}$ < 0.0001, $p_{pSHHtvspG3myc\ =\ NS}$, $p_{pSHHtvspG4}$ < 0.01, $p_{pSHHsvspG3}$ < 0.01, $p_{pSHHsvspG3myc}$ < 0.0001, $p_{pSHHsvspG4}$ < 0.0001, $p_{pG3vsG4}$ = NS, $p_{pG3vspG3myc}$ < 0.0001, $p_{pG4vspG3myc}$ < 0.0001) and **B** Left: Heatmap CCT-complex components abundance across the four MB sugroups Cavalli data (n= 763, WNT=70, SHH=223 ,G4=326,G3=144)[1] Middle : quantification as dotplots for mean per CCT- component per, data are presented as +/- SD and Right: corrplot displaying the pvalues of the t-test results for each comparison based on MB subgroups ($p_{WNTvspSHH}$ = NS, $p_{WNTvsG4}$ < 0.01, $p_{WNTvsG3}$ =0.03, $p_{SHHvsG4}$ = NS , $p_{SHHtvsG3}$ = 0.04, p_{G4vsG3} < 0.0001). . n represents biologically independent human samples.

PCA and heatmap using Nglycan data

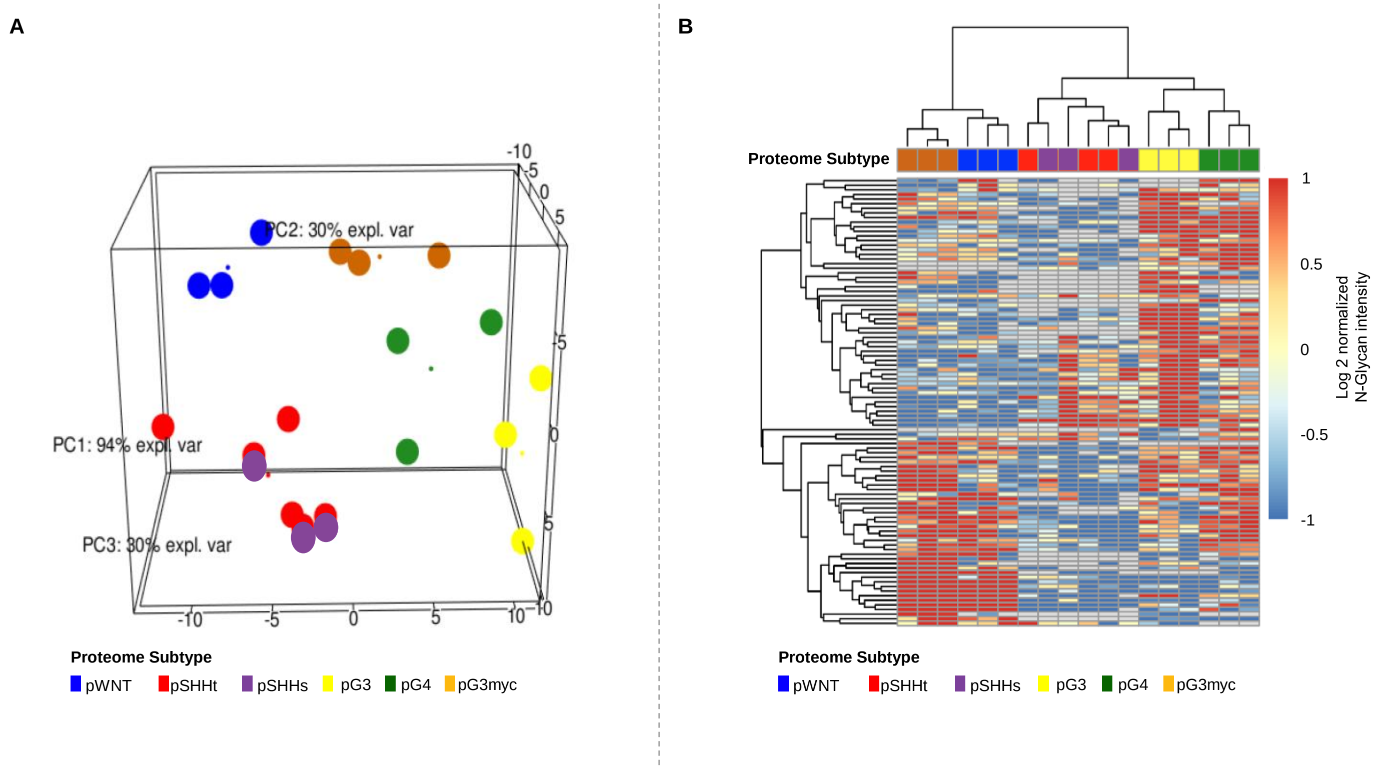

Supplementary Figure 16: A 3D Scatter Plot visualization of the first 3 principal components in NIPALS PCA, based on 302 N-glycans, found in the current FFPE cohort (n_{wnt} =3, n_{pSHHt} =3 n_{pSHHs}=3 n_{pG3} =3 n_{pG3myc} =3 n_{pG4} =3) . Samples were colored according to the proteome subtype. **B** Heatmap visualization of Pearson correlation-based hierarchical clustering with Ward.D linkage, based on 92 ANOVA significant N-glycans between different proteome subtypes of MB. N-glycan abundances have been mean normalized across rows prior to visualization. . n represents biologically independent human samples.

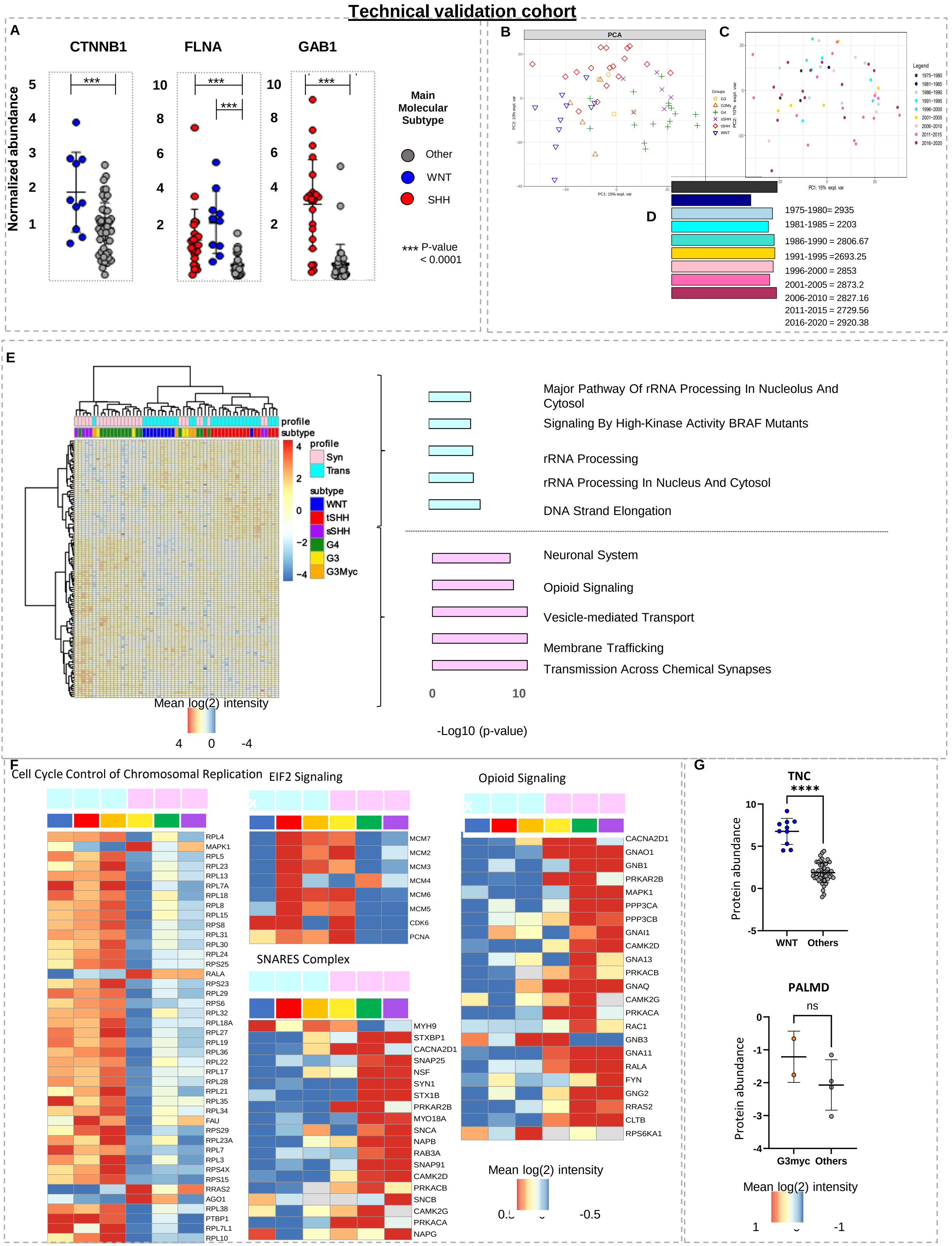

Supplementary Figure 17 : A Housekeeping genes distribution in technical validation cohort(n=57, pWNT=10 , pSHHHt=17, pSHHs=6 ,pG4 = 16, pG3 = 4,G3myc = 4), two-tailed, unpaired-test (CTNNB1: $p_{pWNTvspothers}$ < 0.001, FLNA : $p_{pWNTvspothers}$ < 0.001, : $p_{pSHHvspothers}$ < 0.001, GAB1: $p_{pSHHvspothers}$ < 0.001 **B** PCA reflecting the six proteome subtypes **C** PCA based on sample age **D** number of proteins quantified across samples from different age (mean of samples quantified in the given age range were considered) **E** Hierarchical clustering of samples based on proteome subtypes **F** Enriched pathways in samples belonging to synaptic and transcriptional profiles **G** Biomarkers for pWNT (TNC, n_{pWNT} = 10, n_{others} = 47, : $p_{pWNTvspothers}$ < 0.001) and pG3myc (PALMD,n_{pG3myc} = 2, n_{others} = 4, : $p_{pG3mycvspothers}$ = NS (PALMD was not detected in other samples)) identified in the main cohort. . n represents biologically independent human samples.

Theoretical central dogma of brain tumor biology

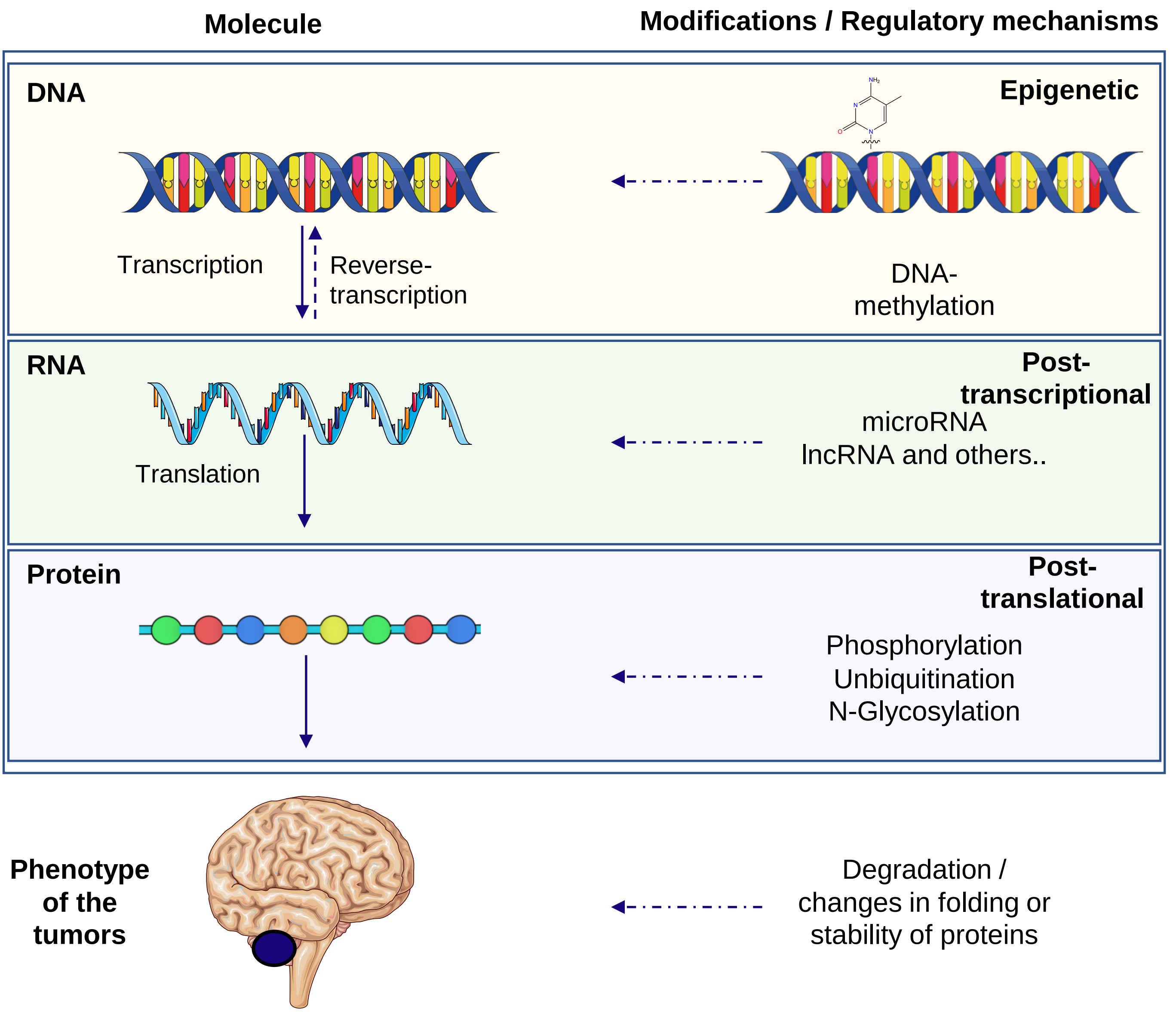

Supplementary Figure 18 : Theoretical central dogma of brain tumor biology
The first level is the DNA (in yellow) :in terms of cancers the most common modification is epigenetic modification. This modification can result in silencing of gene (mostly tumor suppressors) by inhibiting it's transcription to mRNA if methylation occurs at promoter region or can result the gene to be constantly active (mostly oncogenes) if the modification occurs on gene body. The DNA is then transcribed to RNA and hence modifications of the DNA have higher probability of being reflected at the RNA level.

The second level is RNA (in green) : Once the DNA is transcribed to RNA, there can be several post-transcriptional modifications such as inhibition or initiation of translation of mRNA to proteins by several microRNAs, lncRNAs or siRNAs. Thus changes at mRNA level have a higher probability of being detected at the proteome level**.**

The third level is Protein (in light blue) : Here the translated amino acids form disulphide bridges to in turn form proteins which will be responsible for the phenotype of healthy / disease condition. However, the protein structures can also undergo further post-translational modifications such as N-glycosylation or phosphorylation etc, which can indeed affect the stability and structure of proteins. This can result in certain deformed structures which will result in a diseased phenotype.

Hence, not all changes occuring at DNA level will be transmitted to RNA level, and definitely not to the proteome level as there are several steps of modifications or regulations that occur in between.
Similarly, not all changes at RNA level will be reflected at the proteome level due to possible post transcriptional changes. But the proteome level is the final level which is then responsible for the disease or healthy phenotype, and also has the possibility to undergo post translational modifications.

These modifications are known to be more active in case of a disease condition. But it is important to note that some modifications are also essential for normal function and protection from the disease

Parts of art were created with https://bioicons.com/, CC 0 · CC by SA · MIT License:
dna-nucleotides-ribbon icon by Servier https://smart.servier.com/ is licensed under CC-BY 3.0 Unported https://creativecommons.org/licenses/by/3.0/
rna icon by Servier https://smart.servier.com/ is licensed under CC-BY 3.0 Unported https://creativecommons.org/licenses/by/3.0/
Protein_primary_structure icon by DBCLS https://togotv.dbcls.jp/en/pics.html is licensed under CC-BY 4.0 Unported https://creativecommons.org/licenses/by/4.0/
brain-2 icon by Servier https://smart.servier.com/ is licensed under CC-BY 3.0 Unported https://creativecommons.org/licenses/by/3.0/

References

[1] Cavalli FMG, Remke M, Rampasek L, Peacock J, Shih DJH, Luu B, et al. Intertumoral heterogeneity within medulloblastoma subgroups. Cancer Cell. 2017;31:737–54 e736.

Supplementary Table 1

Antibodies and staining programs for immunohstochemistry.

antibody	host/clonality	supplier	catalognumber	lot number	dilution	Ventana program
ALDH1A3	rb, polyclonal	Novus Biologicals	NBP2-15339	A-3	1:1000	CC1st
c-myc	rb, monoclonal	Zeta Corporation	Z2734RL	ZR02042022A-2	1:25	CC2 OptiView
Tenascin c	BC-24, ms monoclonal	Sigma-Aldrich	SAB4200782	107M4823V	1:1000	CC1m
PalmD	rb polyclonal	Novus Bio	NBP2-55156	R31253	1:750	CC1st

Supplementary Table 2

MRM settings per metabolite and internal standard.

Metabolite	Cone [V]	CE [eV]	MRM 12C	MRM ISTD
ALA	14	10	90 > 44	94 > 47
ARG	8	22	175 > 70	185 > 75
ASN	6	14	133 > 74	139 > 77
ASP	4	8	134 > 88	139 > 92
GLN	2	14	147 > 84	154 > 90
GLU	8	12	148 > 102	154 > 107
GLY	18	6	76 > 30	79 > 32
HIS	8	12	156 > 110	165 > 118
ILE	20	16	132 > 69	139 > 74
LEU	20	12	132 > 86	139 > 92
LYS	8	16	147 > 84	155 > 90
MET	18	14	150 > 56	156 > 60
PHE	20	10	166 > 120	176 > 129
PRO	10	8	116 > 70	122 > 75
SER	12	10	106 > 60	110 > 63
THR	2	20	120 > 56	120 > 60
TRP	14	18	205 > 146	218 > 156
TYR	14	28	182 > 91	192 > 98
VAL	14	8	118 > 72	124 > 77

Supplementary Table 3 : All Cohorts included in this study for Proteome Analysis

	Study	Source of the samples	Number of Samples
1.	**Main Cohort (Protein Data) consists of:**<br>• Clinical Information (Supplementary Data 1c)<br>• Protein abundances (Supplementary Data 1a)<br><br>**i) Current study Cohort**<br>➢ Raw Protein abundances (TMT: PXD039319)<br>➢ 80 samples were measured, 10 were standards.<br>➢ 70 samples with mass spectra, however 8 samples had to be excluded due to high blood content, hence **n = 62.**<br><br>**ii) Previously Published cohort included for data integration**<br><br>**PXD006607 (Forget et al. Cohort)**<br>➢ 39 samples were measured, 1 was excluded due to high blood content, hence **n = 38.**<br><br>**MSV000082644 (Archer et al. Cohort)**<br>➢ 50 samples were measured, 5 were standards. Hence **n = 45.**<br><br>**PDC Study Identifier: PDC000180 (Petralia et al. Cohort)**<br>➢ 22 samples were measured, hence **n = 22**	FFPE Medulloblastoma samples of tumors within the years 1976-2022 were obtained from tissue archives from neuropathology units in Munich (Ludwig-Maximilians-University), Heidelberg (University Hospital Heidelberg), Hannover (Hannover Medical School (MHH)), Aachen (RWTH Aachen University Hospital), Augsburg (University of Augsburg) and Hamburg (University Medical Center Hamburg-Eppendorf). Some of these samples were collected as part of the HIT-MED study, which is a registry for developing treatments in children and adolescents with aggressive pediatric brain tumors such as Medulloblastoma and Ependymoma. Some samples (Supplementary Data 1c, Supplementary Data 11) were part of SIOP-PNET5. The present analysis was not a planned SIOP-PNET5-MB study question, but was done from archival material of PNET5-participants from the author's own institution and informed consent of the trial participants. To avoid potential interference with the analysis of SIOP-PNET5-MB trial analyses, the inclusion of these patients was discussed with the PNET5 principal investigator (Stefan Rutkowski) and the analyses were classified not to interfere with predefined SIOP-PNET5-MB study hypotheses. Included PNET5 samples were used for all the analyses in this study, but excluded from survival analysis, since this clinical trial is still not yet published.	$n_{\text{current study main cohort with successful proteome subtype assignment}} = 62$<br>$n_{\text{current study main cohort excluded}} = 8$<br>$n_{\text{Forget et al (PMID: 302050439) with susccessful proteome subtype assignment}} = 38$<br>$n_{\text{Archer et al (PMID: 30205044) with susccessful proteome subtype assignment}} = 45$<br>$n_{\text{Petralia et al (PMID: 33242424) with susccessful proteome subtype assignment}} = 22$
2.	**Biological Validation cohort (Protein Data)**<br>• Clinical Information (Supplementary Data 11)<br>• Protein abundances (Supplementary Data 10a)<br>• Raw Protein abundances (PXD048767)	The validation samples (both technical and biological validation) were a subset from all the samples collected from all the different institutions and HIT-MED.	$n_{\text{biological validation}} = 30$
3.	**Technical Validation cohort**<br>• Clinical information (Supplementary Data 1c)<br>• Protein abundances (Supplementary Data 10g)<br>• Raw Protein abundances (PXD048767)	The validation samples (both technical and biological validation) were a subset from all the samples collected from all the different institutions and HIT-MED.	$n_{\text{technical validation}} = 57$
