# Supplementary material for: Multiomic profiling of medulloblastoma reveals subtype-specific targetable alterations at the proteome and N-glycan level
Source: Nat Commun. 2024 Jul 24;15:6237. doi: 10.1038/s41467-024-50554-z (PMC11266559; doi:10.1038/s41467-024-50554-z)
Supplement: Supplementary file 3 — Description of Additional Supplementary Files [file 41467_2024_50554_MOESM3_ESM.pdf]

Description of Additional Supplementary Files

Supplementary Data 1:

Supplementary Data 1a: Harmonized Protein abundances across all considered studies. log 2 transformed, median normalized and centered protein abundances after HarmonizR usage (ComBat, parametric, L/S Scaling) for data integration and harmonisazion of independent proteome datasets.

Supplementary Data 1b: Considered Proteins for the characterization of proteome subtypes. log 2 transformed, median normalized and centered protein abundances after HarmonizR usage (ComBat, parametric, L/S Scaling) for all proteins found in >= 30% of all defined proteome subtypes (pWNT, pSHHt, pSHHs, pG3, pG4, pG3myc)

Supplementary Data 1c: Clinical sample information

Supplementary Data 1d: M-Values

Supplementary Data 2:

Supplementary Data 2a: ANOVA test results between proteome groups. log 2 transformed, median normalized and centered protein abundances after HarmonizR usage (ComBat, parametric, L/S Scaling) for all proteins found in >30% of all defined proteome subtypes (pWNT, pSHHt, pSHHs, pG3, pG4, pG3myc). ANOVA Testing was performed with a p-value cutoff of 0.05

Supplementary Data 3:

Supplementary Data 3a: Significantly enriched GO-BP Terms in the replicative/transcriptional profile (pSHHt, pG3myc, pWNT). Gene Set Enrichment Analysis was performed by using the GSEA software (version 4.1, Broad Institute, San Diego, CA, USA), (Z) 1000 permutations were used. Permutation was performed based on gene sets. A weighted enrichment statistic was applied, using the signal-to-noise ratio as a metric for ranking genes. No additional normalization was applied within GSEA. Gene sets smaller than 15 and bigger than 500 genes were excluded from analysis. Gene sets, identified using differential expression analysis at an FDR < 0.25 and a p-value < 0.1. were considered further.

Supplementary Data 3b: Significantly enriched GO-BP Terms in the synaptic profile (pSHHs, pG4, pG3). Gene Set Enrichment Analysis was performed by using the GSEA software (version 4.1, Broad Institute, San Diego, CA, USA), (Z) 1000 permutations were used. Permutation was performed based on gene sets. A weighted enrichment statistic was applied, using the signal-to-noise ratio as a metric for ranking genes. No additional normalization was applied within GSEA. Gene sets smaller than 15 and bigger than 500 genes were excluded from analysis. Gene sets, identified using differential expression analysis at an FDR < 0.25 and a p-value < 0.1. were considered further.

Supplementary Data 3c: IPA Analysis results - Info

Supplementary Data 3d: Drugs associated with cell cycle control of chromosomal replication - Arranged as drugs related to Medulloblastoma, drugs related to brain tumors and drugs related to cancer. Additionally, the list of proteins that make up the respective signalling pathways

Supplementary Data 3e: Drugs associated with EIF2 signalling - Arranged as drugs related to Medulloblastoma, drugs related to brain tumors and drugs related to cancer. Additionally, the list of proteins that make up the respective signalling pathways

Supplementary Data 3f: Drugs associated with Opiod signaling - Arranged as drugs related to Medulloblastoma, drugs related to brain tumors and drugs related to cancer. Additionally, the list of proteins that make up the respective signalling pathways

Supplementary Data 3g: Drugs associated with SNARE complex - Arranged as drugs related to Medulloblastoma, drugs related to brain tumors and drugs related to cancer. Additionally, the list of proteins that make up the respective signalling pathways

Supplementary Data 3h: Common pathways between FFPE, Forget et al, 2017, Archer et al, 2018, Petralia et al ,2020 - Unpaired T-test was performed between the samples belonging to syaptic and transcriptional profile. Samples were allocated to respective profiles based on their sepration in the PCA. Proteins with log2FC > 1.5 and p-value < 0.05 were used for REACTOME based pathway analysis in EnrichR. Common pathways from all datasets are displayed

Supplementary Data 4:

Supplementary Data 4a: Correlation values for TOP biomarker : Biomarkers correlating with any CpG site of any gene and crossing the pearson correlation threshold of 0.7 and Biomarkers correlating with the CpG site of their own gene and crossing the threshold of 0.7 Pearson correlation between 381717 CpG sites and 3990 proteins in non-subtype specific manner and applying a cutoff of 0.7 shows the correlation of proteins with CpG sit for any gene and the table in green shows the proteins which correlate with the CpG site of their own gene and cross the threshold of 0.7

Supplementary Data 4b: Group specific correlation > 0.7 for pWNT: Pearson correlation between 381717 CpG sites and 3990 proteins in for pWNT samples and applying a cutoff of 0.7 and matching the proteins to their respective genes CpG site, additionally a table giving all the information about this cpf site has also been added

Supplementary Data 4c: Group specific correlation > 0.7 for pSHHs: Pearson correlation between 381717 CpG sites and 3990 proteins in for pSHHs samples and applying a cutoff of 0.7 and matching the proteins to their respective genes CpG site. Additionally, a table giving further information for this correlating CpG site has also been added

Supplementary Data 4d: Group specific correlation > 0.7 for pSHHt: Pearson correlation between 381717 CpG sites and 3990 proteins in for pSHHt samples and applying a cutoff of 0.7 and matching the proteins to their respective genes CpG site. Additionally, a table giving further information for this correlating CpG site has also been added

Supplementary Data 4e: Group specific correlation > 0.7 for pG3: Pearson correlation between 381717 CpG sites and 3990 proteins in for pG3 samples and applying a cutoff of 0.7 and matching the proteins to their respective genes CpG site. Additionally, a table giving further information for this correlating CpG site has also been added

Supplementary Data 4f : Group specific correlation > 0.7 for pG4: Pearson correlation between 381717 CpG sites and 3990 proteins in for pG4 samples and applying a cutoff of 0.7 and matching the proteins to their respective genes CpG site. Additionally, a table giving further information for this correlating CpG site has also been added

Supplementary Data 4g: Group specific correlation > 0.7 for pG3myc: Pearson correlation between 381717 CpG sites and 3990 proteins in for pG3myc samples and applying a cutoff of 0.7 and matching the proteins to their respective genes CpG site. Additionally, a table giving further information for this correlating CpG site has also been added

Supplementary Data 4h: mixOmics plot loadings and circus plot correlation values

Supplementary Data 5:

Supplementary Data 5a: Students T-test results between pSHHs and all other groups. log 2 transformed, median normalized and centered protein abundances after HarmonizR usage (ComBat, parametric, L/S Scaling) for all proteins found in >30% of all defined proteome subtypes (pWNT, pSHHt, pSHHs, pG3, pG4, pG3myc). Proteins identified with a p-value < 0.05 and at least 1.5-fold difference between pSHHs and all other groups were considered significantly differential abundant

Supplementary Data 5b: Students T-test results between pSHHt and all other groups. log 2 transformed, median normalized and centered protein abundances after HarmonizR usage (ComBat, parametric, L/S Scaling) for all proteins found in >30% of all defined proteome subtypes (pWNT, pSHHt, pSHHs, pG3, pG4, pG3myc). Proteins identified with a p-value < 0.05 and at least 1.5-fold difference between pSHHt and all other groups were considered significantly differential abundant

Supplementary Data 5c: Significantly enriched REACTOME Terms in pSHHs, compared to all other groups. Gene Set Enrichment Analysis was performed by using the GSEA software (version 4.1, Broad Institute, San Diego, CA, USA), (Z) 1000 permutations were used. Permutation was performed based on gene sets. A weighted enrichment statistic was applied, using the signal-to-noise ratio as a metric for ranking genes. No additional normalization was applied within GSEA. Gene sets smaller than 15 and bigger than 500 genes were excluded from analysis. Gene sets, identified using differential expression analysis at an FDR < 0.25 and a p-value < 0.1. were considered further.

Supplementary Data 5d: Significantly enriched REACTOME Terms in all other groups, compared to pSHHs Gene Set Enrichment Analysis was performed by using the GSEA software (version 4.1, Broad Institute, San Diego, CA, USA), (Z) 1000 permutations were used. Permutation was performed based on gene sets. A weighted enrichment statistic was applied, using the signal-to-noise ratio as a metric for ranking genes. No additional normalization was applied within GSEA. Gene sets smaller than 15 and bigger than 500 genes were excluded from analysis. Gene sets, identified using differential expression analysis at an FDR < 0.25 and a p-value < 0.1. were considered further.

Supplementary Data 5e: Significantly enriched REACTOME Terms in pSHHt, compared to all other groups. Gene Set Enrichment Analysis was performed by using the GSEA software (version 4.1, Broad Institute, San Diego, CA, USA), (Z) 1000 permutations were used. Permutation was performed based on gene sets. A weighted enrichment statistic was applied, using the signal-to-noise ratio as a metric for ranking genes. No additional normalization was applied within GSEA. Gene sets smaller than 15 and bigger than 500 genes were excluded from analysis. Gene sets, identified using differential expression analysis from GSEA at an FDR < 0.25 and a p-value < 0.1. were considered further.

Supplementary Data 5f : Significantly enriched REACTOME Terms in all other groups, compared to pSHHt Gene Set Enrichment Analysis was performed by using the GSEA software (version 4.1, Broad Institute, San Diego, CA, USA), (Z) 1000 permutations were used. Permutation was performed based on gene sets. A weighted enrichment statistic was applied, using the signal-to-noise ratio as a metric for ranking genes. No additional normalization was applied within GSEA. Gene sets smaller than 15 and bigger than 500 genes were excluded from analysis. Gene sets, identified using differential expression analysis at an FDR < 0.25 and a p-value < 0.1. were considered further.

Supplementary Data 5g: Loadings CNV Plots pSHHt for methylation data: raw idats files were read into conumee and preprocessIllumina normalized. The mean segmentation values for default bin sizes after performing circular binary segmentation were then used for calculating the frequency of gains and losses in CNApp web Tool (cutoff for gain or loss was value > |0.2|) Loadings CNV Plots pSHHt For proteome data : Copynumber package was used to generate the segmentation values for proteome data and loaded into CNApp web Tool to calculate the frequency of gains or losses (cutoff for gain or loss was value > |0.2|)

Supplementary Data 5h: Loadings CNV Plots pSHHs for methylation data: raw idats files were read into conumee and preprocessIllumina normalized. The mean segmentation values for default bin sizes after performing circular binary segmentation were then used for calculating the frequency of gains and losses in CNApp web Tool (cutoff for gain or loss was value > |0.2|) Loadings CNV Plots pSHHs For proteome data : Copynumber package was used to generate the segmentation values for proteome data and loaded into CNApp web Tool to calculate the frequency of gains or losses (cutoff for gain or loss was value > |0.2|)

Supplementary Data 5i : Students T-test results between pSHHt (TP53 Mut) and pSHHt (TP53 WT). log 2 transformed, median normalized and centered protein abundances after HarmonizR usage (ComBat, parametric, L/S Scaling) for all proteins found in >30% of all defined proteome subtypes (pWNT, pSHHt, pSHHs, pG3, pG4, pG3myc). Proteins identified with a p-value < 0.05 and at least 1.5-fold difference between (TP53 Mut) and pSHHt (TP53 WT) were considered significantly differential abundant

Supplementary Data 5j: Validation cohort information-Reference Cases (Waszak et al. 2020)

Supplementary Data 5k: Validation cohort information- SHH Cases (Waszak et al. 2020)

Supplementary Data 5l: Processed Amino Acid data for SHH samples and ManWhitneyTest results fo r amino acids between pSHHs and pSHHt. Amino acids with a p-value < 0.05 were considered significant and are marked in green

Supplementary Data 5m: Processed Metabolite data for SHH samples and ManWhitneyTest results for metabolites between pSHHs and pSHHt. Metabolites with a p-value < 0.05 were considered significant and are marked in green

Supplementary Data 6:

Supplementary Data 6a: Students T-test results between pG3myc and all other groups. log 2 transformed, median normalized and centered protein abundances after HarmonizR usage (ComBat, parametric, L/S Scaling) for all proteins found in >30% of all defined proteome subtypes (pWNT, pSHHt, pSHHs, pG3, pG4, pG3myc). Proteins identified with a p-value < 0.05 and at least 1.5-fold difference between pG3myc and all other groups were considered significantly differential abundant

Supplementary Data 6b: Students T-test results between pG3 and all other groups. log 2 transformed, median normalized and centered protein abundances after HarmonizR usage (ComBat, parametric, L/S Scaling) for all proteins found in >30% of all defined proteome subtypes (pWNT, pSHHt, pSHHs, pG3, pG4, pG3myc). Proteins identified with a p-value < 0.05 and at least 1.5-fold difference between pG3 and all other groups were considered significantly differential abundant

Supplementary Data 6c: Students T-test results between pG4 and all other groups. log 2 transformed, median normalized and centered protein abundances after HarmonizR usage (ComBat, parametric, L/S Scaling) for all proteins found in >30% of all defined proteome subtypes (pWNT, pSHHt, pSHHs, pG3, pG4, pG3myc). Proteins identified with a p-value < 0.05 and at least 1.5-fold difference between pG4 and all other groups were considered significantly differential abundant

Supplementary Data 6d: Significantly enriched REACTOME Terms in pG3myc, compared to all other groups. Gene Set Enrichment Analysis was performed by using the GSEA software (version 4.1, Broad Institute, San Diego, CA, USA), (Z) 1000 permutations were used. Permutation was performed based on gene sets. A weighted enrichment statistic was applied, using the signal-to-noise ratio as a metric for ranking genes. No additional normalization was applied within GSEA. Gene sets smaller than 15 and bigger than 500 genes were excluded from analysis. Gene sets, identified using differential expression analysis at an FDR < 0.25 and a p-value < 0.1. were considered further.

Supplementary Data 6e: Significantly enriched REACTOME Terms in all other groups, compared to pG3myc Gene Set Enrichment Analysis was performed by using the GSEA software (version 4.1, Broad Institute, San Diego, CA, USA), (Z) 1000 permutations were used. Permutation was performed based on gene sets. A weighted enrichment statistic was applied, using the signal-to-noise ratio as a metric for ranking genes. No additional normalization was applied within GSEA. Gene sets smaller than 15 and bigger than 500 genes were excluded from analysis. Gene sets, identified using differential expression analysis at an FDR < 0.25 and a p-value < 0.1. were considered further.

Supplementary Data 6f : Significantly enriched REACTOME Terms in pG3, compared to all other groups. Gene Set Enrichment Analysis was performed by using the GSEA software (version 4.1, Broad Institute, San Diego, CA, USA), (Z) 1000 permutations were used. Permutation was performed based on gene sets. A weighted enrichment statistic was applied, using the signal-to-noise ratio as a metric for ranking genes. No additional normalization was applied within GSEA. Gene sets smaller than 15 and bigger

than 500 genes were excluded from analysis. Gene sets, identified using differential expression analysis at an FDR < 0.25 and a p-value < 0.1. were considered further.

Supplementary Data 6g: Significantly enriched REACTOME Terms in all other groups, compared to pG3 Gene Set Enrichment Analysis was performed by using the GSEA software (version 4.1, Broad Institute, San Diego, CA, USA), (Z) 1000 permutations were used. Permutation was performed based on gene sets. A weighted enrichment statistic was applied, using the signal-to-noise ratio as a metric for ranking genes. No additional normalization was applied within GSEA. Gene sets smaller than 15 and bigger than 500 genes were excluded from analysis. Gene sets, identified using differential expression analysis at an FDR < 0.25 and a p-value < 0.1. were considered further.

Supplementary Data 6h: Significantly enriched REACTOME Terms in pG4, compared to all other groups. Gene Set Enrichment Analysis was performed by using the GSEA software (version 4.1, Broad Institute, San Diego, CA, USA), (Z) 1000 permutations were used. Permutation was performed based on gene sets. A weighted enrichment statistic was applied, using the signal-to-noise ratio as a metric for ranking genes. No additional normalization was applied within GSEA. Gene sets smaller than 15 and bigger than 500 genes were excluded from analysis. Gene sets, identified using differential expression analysis at an FDR < 0.25 and a p-value < 0.1. were considered further.

Supplementary Data 6i : Significantly enriched REACTOME Terms in all other groups, compared to pG4 Gene Set Enrichment Analysis was performed by using the GSEA software (version 4.1, Broad Institute, San Diego, CA, USA), (Z) 1000 permutations were used. Permutation was performed based on gene sets. A weighted enrichment statistic was applied, using the signal-to-noise ratio as a metric for ranking genes. No additional normalization was applied within GSEA. 1000 permutations Gene sets smaller than 15 and bigger than 500 genes were excluded from analysis. Gene sets, identified using differential expression analysis at an FDR < 0.25 and a p-value < 0.1. were considered further.

Supplementary Data 6j: Loadings CNV Plots pG3myc For methylation data : raw idats files were read into conumee and preprocessIllumina normalized. The mean segmentation values for default bin sizes after performing circular binary segmentation were then used for calculating the frequency of gains and losses in CNApp web Tool (cutoff for gain or loss was value > |0.2|) Loadings CNV Plots pG3myc For proteome data : Copynumber package was used to generate the segmentation values for proteome data and loaded into CNApp web Tool to calculate the frequency of gains or losses (cutoff for gain or loss was value > |0.2|)

Supplementary Data 6k: Loadings CNV Plots pG3 For methylation data : raw idats files were read into conumee and preprocessIllumina normalized. The mean segmentation values for default bin sizes after performing circular binary segmentation were then used for calculating the frequency of gains and losses in CNApp web Tool (cutoff for gain or loss was value > |0.2|) Loadings CNV Plots pG3 For proteome data : Copynumber package was used to generate the segmentation values for proteome data and loaded into CNApp web Tool to calculate the frequency of gains or losses (cutoff for gain or loss was value > |0.2|)

Supplementary Data 6l: Loadings CNV Plots pG4 For methylation data : raw idats files were read into conumee and preprocessIllumina normalized. The mean segmentation values for default bin sizes after performing circular binary segmentation were then used for calculating the frequency of gains and losses in CNApp web Tool (cutoff for gain or loss was value > |0.2|)

Loadings CNV Plots pG4 For proteome data : Copynumber package was used to generate the segmentation values for proteome data and loaded into CNApp web Tool to calculate the frequency of gains or losses (cutoff for gain or loss was value > |0.2|)

Supplementary Data 6m: Significantly enriched REACTOME Terms in pG3 and pG4, compared to pG3myc Gene Set Enrichment Analysis was performed by using the GSEA software (version 4.1, Broad Institute, San Diego, CA, USA), (Z) 1000 permutations were used. Permutation was performed based on gene sets. A weighted enrichment statistic was applied, using the signal-to-noise ratio as a metric for ranking genes. No additional normalization was applied within GSEA. Gene sets smaller than 15 and bigger than 500 genes were excluded from analysis. Gene sets, identified using differential expression analysis at an FDR < 0.25 and a p-value < 0.1. were considered further.

Supplementary Data 7:

Supplementary Data 7a: Students T-test results between pWNT and all other groups. log 2 transformed, median normalized and centered protein abundances after HarmonizR usage (ComBat, parametric, L/S Scaling) for all proteins found in >30% of all defined proteome subtypes (pWNT, pSHHt, pSHHs, pG3, pG4, pG3myc). Proteins identified with a p-value < 0.05 and at least 1.5-fold difference between pWNT and all other groups were considered significantly differential abundant

Supplementary Data 7b: Significantly enriched REACTOME Terms in all other groups, compared to pWNT. Gene Set Enrichment Analysis was performed by using the GSEA software (version 4.1, Broad Institute, San Diego, CA, USA), (Z) 1000 permutations were used. Permutation was performed based on gene sets. A weighted enrichment statistic was applied, using the signal-to-noise ratio as a metric for ranking genes. No additional normalization was applied within GSEA. Gene sets smaller than 15 and bigger than 500 genes were excluded from analysis. Gene sets, identified using differential expression analysis at an FDR < 0.25 and a p-value < 0.1. were considered further.

Supplementary Data 7c: Significantly enriched REACTOME Terms in pWNT, compared to all other groups. Gene Set Enrichment Analysis was performed by using the GSEA software (version 4.1, Broad Institute, San Diego, CA, USA), (Z) 1000 permutations were used. Permutation was performed based on gene sets. A weighted enrichment statistic was applied, using the signal-to-noise ratio as a metric for ranking genes. No additional normalization was applied within GSEA. Gene sets smaller than 15 and bigger than 500 genes were excluded from analysis. Gene sets, identified using differential expression analysis at an FDR < 0.25 and a p-value < 0.1. were considered further.

Supplementary Data 7d: Loadings CNV Plots pWNT For methylation data : raw idats files were read into conumee and preprocessIllumina normalized. The mean segmentation values for default bin sizes after performing circular binary segmentation were then used for calculating the frequency of gains and losses in CNApp web Tool (cutoff for gain or loss was value > |0.2|)
Loadings CNV Plots pWNT For proteome data : Copynumber package was used to generate the segmentation values for proteome data and loaded into CNApp web Tool to calculate the frequency of gains or losses (cutoff for gain or loss was value > |0.2|)

Supplementary Data 8:

Supplementary Data 8a: Students T-test results between pWNT andpG3myc. log 2 transformed,median normalized and centered protein abundances after HarmonizR usage (ComBat, parametric, L/S Scaling) for all proteins found in >30% of all defined proteome subtypes (pWNT, pSHHt, pSHHs, pG3, pG4, pG3myc). Proteins identified with a p-value < 0.05 and at least 1.5 fold difference between pSHHs and all other groups were considered significantly differential abundant

Supplementary Data 8b: Significantly enriched REACTOME Terms in pWNT, compared to pG3myc.
Gene Set Enrichment Analysis was performed by using the GSEA software (version 4.1, Broad Institute, San Diego, CA, USA), (Z) 1000 permutations were used. Permutation was performed based on gene sets. A weighted enrichment statistic was applied, using the signal-to-noise ratio as a metric for ranking genes. No additional normalization was applied within GSEA. Gene sets smaller than 15 and bigger than 500 genes were excluded from analysis. . Gene sets, identified at an FDR < 0.25 and a p-value < 0.1. were considered further.

Supplementary Data 8c: Significantly enriched REACTOME Terms in pG3myc compared to pWNT
Gene Set Enrichment Analysis was performed by using the GSEA software (version 4.1, Broad Institute, San Diego, CA, USA), (Z) 1000 permutations were used. Permutation was performed based on gene sets. A weighted enrichment statistic was applied, using the signal-to-noise ratio as a metric for ranking genes. No additional normalization was applied within GSEA. Gene sets smaller than 15 and bigger than 500 genes were excluded from analysis. . Gene sets, identified at an FDR < 0.25 and a p-value < 0.1. were considered further.

Supplementary Data 8d: Pearson correlation for CCT complex components across the three omics (for matched samples, n = 60)

Supplementary Data 9:

Supplementary Data 9a: Unnormalized N-Glycan abundances for all analyzed samples (N=3 per proteome group).Blue:pWNT, .Purple: pSHHs, Red:pSHHt, Green:pG4, Yellow: pG3, Orange: pG3myc

Supplementary Data 9b: ANOVA Test results between all proteomic groups (pWNT, pSHHs, pSHHt, pG3, pG4 and pG3myc) at the N-Glycan level. Log2 transformed, median normalized N-Glycan abundances of 303 quantified N-Glycans were used. N-Glycans identified with a p-value <0.05 were as considered significantly differential abundant.

Supplementary Data 10:

Supplementary Data 10a: Harmonized Protein abundances for Biological validation samples. log 2 transformed, median normalized and centered protein abundances after HarmonizR usage (ComBat, parametric, L/S Scaling)

Supplementary Data 10b: Considered Proteins for the characterization of proteome subtypes. log 2 transformed, median normalized and centered protein abundances after HarmonizR usage (ComBat, parametric, L/S Scaling) for all proteins found in >= 70% of all defined proteome subtypes (pWNT, pSHHt, pSHHs, pG3, pG4, pG3myc)

Supplementary Data 10c: Mean pearson correlation between main cohort and biological validation proteome subtype samples

Supplementary Data 10d: Student T-test between the samples belonging to synaptic and transcriptional profile. LogFC >= abs (1.5), adj.pvalue < 0.01, up = up regulated in Synaptic profile samples and down = up regulated in transcriptional profile samples

Supplementary Data 10e: Pathways Enriched in the samples belonging to the Synaptic profile. EnrichR, Reactome 2022 database, using all proteins, unpaired t-test, logFC >= abs(1.5) and adj.pvalue < 0.01

Supplementary Data 10f: Pathways Enriched in the samples belonging to the Transcriptional profile. EnrichR, Reactome 2022 database, using all proteins, unpaired t-test, with logFC >= abs(1.5) and adj.pvalue < 0.01

Supplementary Data 10g: Harmonized Protein abundances for Technical validation samples. log 2 transformed, median normalized and centered protein abundances after HarmonizR usage (ComBat, parametric, L/S Scaling)

Supplementary Data 11:

Supplementary Data 11: Clinical sample information for biological validation samples
