# Supplementary material for: Multiomic profiling of medulloblastoma reveals subtype-specific targetable alterations at the proteome and N-glycan level
Source: Nat Commun. 2024 Jul 24;15:6237. doi: 10.1038/s41467-024-50554-z (PMC11266559; doi:10.1038/s41467-024-50554-z)
Supplement: Supplementary file 15 — Reporting Summary [file 41467_2024_50554_MOESM15_ESM.pdf]

nature portfolio

Corresponding author(s): Julia E. Neumann

Last updated by author(s): June 18 6 2024

Reporting Summary

Nature Portfolio wishes to improve the reproducibility of the work that we publish. This form provides structure for consistency and transparency in reporting. For further information on Nature Portfolio policies, see our Editorial Policies and the Editorial Policy Checklist.

Statistics

For all statistical analyses, confirm that the following items are present in the figure legend, table legend, main text, or Methods section.

n/a	Confirmed	
☐	☒	The exact sample size (*n*) for each experimental group/condition, given as a discrete number and unit of measurement
☐	☒	A statement on whether measurements were taken from distinct samples or whether the same sample was measured repeatedly
☐	☒	The statistical test(s) used AND whether they are one- or two-sided<br>*Only common tests should be described solely by name; describe more complex techniques in the Methods section.*
☐	☒	A description of all covariates tested
☐	☒	A description of any assumptions or corrections, such as tests of normality and adjustment for multiple comparisons
☐	☒	A full description of the statistical parameters including central tendency (e.g. means) or other basic estimates (e.g. regression coefficient) AND variation (e.g. standard deviation) or associated estimates of uncertainty (e.g. confidence intervals)
☐	☒	For null hypothesis testing, the test statistic (e.g. F, t, r) with confidence intervals, effect sizes, degrees of freedom and P value noted<br>*Give P values as exact values whenever suitable.*
☒	☐	For Bayesian analysis, information on the choice of priors and Markov chain Monte Carlo settings
☐	☒	For hierarchical and complex designs, identification of the appropriate level for tests and full reporting of outcomes
☐	☒	Estimates of effect sizes (e.g. Cohen's *d*, Pearson's *r*), indicating how they were calculated

Our web collection on statistics for biologists contains articles on many of the points above.

Software and code

Policy information about availability of computer code

Data collection	No specific Software was used for data collection.
Data analysis	R (Version 4.0.5).,minfi package (Version 1.36.0),limma package (Version 3.46.0),ComplexHeatmap (Version 2.6.2),pheatmap (Version 1.0.12),MaxQuant software (Max Plank Institute for Biochemistry, Version 1.6.2.10),Xcalibur Qual Browser (Version No 4.2.28.14),Skyline software (Version No 21.1.0.278),HarmonizR (Version 0.0.0.9),Perseus software (Max Plank Institute for Biochemistry, Version 1.5.8.5),mixOmics package (Version 6.19.4.),Bioconductor (version 3.14),ConsensusClusterPlus package (Version 1.6),PRISM (GraphPad, Version 5) ,Microsoft excel (Version 16.5.),GSEA software (version 4.1, Broad Institute, San Diego, CA, USA), EnrichmentMap (version 3.3) ,AutoAnnotate (version 1.3) ,minfiData package (Version 0.36.0),minfidataEPIC (Version1.16.0),Conumee package (Version 1.24.0),CNAppWeb tool (Shiny R package (version 1.1.0)),. Copynumber (Version 1.30.0) ,anaconda JupyterLab (Version 3.0.14),NDP view v2.7.43 software,Adobe Photoshop 2022 (Adobe Inc., San Jose, USA),

For manuscripts utilizing custom algorithms or software that are central to the research but not yet described in published literature, software must be made available to editors and reviewers. We strongly encourage code deposition in a community repository (e.g. GitHub). See the Nature Portfolio guidelines for submitting code & software for further information.

Data

Policy information about availability of data

All manuscripts must include a data availability statement. This statement should provide the following information, where applicable:

- Accession codes, unique identifiers, or web links for publicly available datasets
- A description of any restrictions on data availability
- For clinical datasets or third party data, please ensure that the statement adheres to our policy

Proteome data have been deposited under PXD039319 (TMT data) [https://www.ebi.ac.uk/pride/archive/projects/PXD039319], and PXD048767 (validation cohorts) [https://www.ebi.ac.uk/pride/archive/projects/PXD048767]. DNA Methylation and RNA Seq data can be accessed via GSE243796 [https://www.ncbi.nlm.nih.gov/geo/query/acc.cgi?acc=GSE243796] containing subsets GSE222478 (450K array DNA methylation data) [https://www.ncbi.nlm.nih.gov/geo/query/acc.cgi?acc=GSE222478], GSE243768 (EPIC array DNA methylation data) [https://www.ncbi.nlm.nih.gov/geo/query/acc.cgi?acc=GSE243768] and GSE243795 (RNA seq data) [https://www.ncbi.nlm.nih.gov/geo/query/acc.cgi?acc=GSE243795]. Metabolomics and amino acid data have been deposited to the EMBL-EBI MetaboLights database84 with the identifier MTBLS9830 [https://www.ebi.ac.uk/metabolights/MTBLS9830] and MTBLS9836 [https://www.ebi.ac.uk/metabolights/MTBLS9836] respectively. Glycan data has been deposited at GlycoPOST106 with the identifier GPST000414 [https://glycopost.glycosmos.org/entry/GPST000414]. Previously published data were included from EGAS00001001953 [https://ega-archive.org/studies/EGAS00001002953], from GSE10472817 [https://www.ncbi.nlm.nih.gov/geo/query/acc.cgi?acc=GSE104728], GSE13005180 [https://www.ncbi.nlm.nih.gov/geo/query/acc.cgi?acc=GSE130051], GPL222865 [https://www.ncbi.nlm.nih.gov/geo/query/acc.cgi?acc=GPL22286], MSV000082644 (MassIVE online repository) [https://www.omicsdi.org/dataset/massive/MSV000082644] and PXD006607 [https://www.ebi.ac.uk/pride/archive/projects/PXD006607], PXD01683230 [https://www.ebi.ac.uk/pride/archive/projects/PXD016832], or through the Clinical Proteomic Tumor Analysis Consortium Data Portal [https://cptac-data-portal.georgetown.edu/cptacPublic/] and the Proteomics Data Commons [https://pdc.cancer.gov/pdc/]. Source data are provided with this paper.

Human research participants

Policy information about studies involving human research participants and Sex and Gender in Research.

Reporting on sex and gender	All patient characteristics are included in the published article under supplementary data file 1 and 11.
Population characteristics	All relevant population characteristics are included in the published article under supplementary data file 1 and 11.
Recruitment	FFPE Medulloblastoma samples of tumors within the years 1976-2021 were obtained from tissue archives from various neuropathology units in Germany including cases that had been collected within the HIT-MED study cohort.
Ethics oversight	This research complies with all relevant ethical regulations. Investigations were performed in accordance with local and national ethical rules of patient's material and have, therefore, been performed in accordance with the ethical standards laid down in an appropriate version of the 1964 Declaration of Helsinki. Ethics approval was waived by the Ethics Committee of the Hamburg Chamber of Physicians. All patients gave their informed consent for scientific use of the data. All samples underwent anonymization. Single patients were enrolled in the SIOP PNET5 study of the HIT-MED study center (for details see Supplementary data file 1c, Supplementary data file 11, clinical data for these patients was excluded from further analysis).

Note that full information on the approval of the study protocol must also be provided in the manuscript.

Field-specific reporting

Please select the one below that is the best fit for your research. If you are not sure, read the appropriate sections before making your selection.

☒ Life sciences ☐ Behavioural & social sciences ☐ Ecological, evolutionary & environmental sciences

For a reference copy of the document with all sections, see nature.com/documents/nr-reporting-summary-flat.pdf

Life sciences study design

All studies must disclose on these points even when the disclosure is negative.

Sample size	No sample size calculation was performed. As medulloblastoma is a rare brain tumor, samples were included based on availability of material but at least 9 samples per known main molecular subgroup of medulloblastoma were included in our FFPE dataset. To ensure large sample sizes for statistical tests, our in house data was integrated with available proteome data from published cohorts. Consensus clustering revealed 6 stable subclusters that were used for further downstream analyses.<br>For proteome data acquisition, a total number 176 patients was analyzed by LC-MS/MS, representing the four main molecular subtypes of Medulloblastoma (Supplementary Table 1). In the current FFPE study 71 patients were included. 105 cases were obtained from public repositories. For N-Glycan data, three randomly selected samples (with sufficient material) for each predefined proteome subtype were analyzed (N=18).<br>DNA methylome analysis was performed on 122 samples out of which 42 were measured in-house on Illumina Infinium 850K arrays, 80 samples were obtained from previously published datasets.
Data exclusions	For in-house proteome data, 9 patients were excluded due to high blood protein yields in LC-MS/MS spectra. Associated raw spectra for excluded samples can be accessed through the ProteomeXchange Consortium via the PRIDE partner repository with the dataset identifier

	PXD039319. Excluded samples are highlighted in supplementary table 1c. Multi-omics analysis was only performed on samples for which both methylome and proteome data was available. No further data points were excluded for proteome, DNA-Methylation and RNA-Sequencing data respecitvely.
Replication	For publicly available datasets the availability and number of replicates depended on the respective experimental setup given in the original study. For in house generated data each main molecular subtype of MB was analyzed in at least 9 biological replicates, depending on the number of available tissue secions. Technical validation of n=57 tumors confirmed results of the main cohort (see Supplementary Figure 17). Additionally a biological validation cohort was used that confirmed results again (n=31, Figure 10).
Randomization	In-house LC-MS/MS measurements (Proteome and N-Glycan data) as well as in house DNA-Methylation data acquisition was randomized across the main molecular subtypes of Medulloblastoma. For publicly available datasets randomization depended on the respective experimental setup given in the original study.TMT based MS-studies used randominazition across molecular subtypes/bain tumor entities for batches. For non TMT-based studies this was not relevant as now batched occured.
Blinding	The identification of proteome subtypes of MB was performed in a blinded manner, independent of clinical variables, listed in supplementary table 1c.

Reporting for specific materials, systems and methods

We require information from authors about some types of materials, experimental systems and methods used in many studies. Here, indicate whether each material, system or method listed is relevant to your study. If you are not sure if a list item applies to your research, read the appropriate section before selecting a response.

Materials & experimental systems

n/a	Involved in the study
☐	☒ Antibodies
☐	☒ Eukaryotic cell lines
☒	☐ Palaeontology and archaeology
☒	☐ Animals and other organisms
☐	☒ Clinical data
☒	☐ Dual use research of concern

Methods

n/a	Involved in the study
☒	☐ ChIP-seq
☒	☐ Flow cytometry
☒	☐ MRI-based neuroimaging

Antibodies

Antibodies used

Antibody: ALDH1A3
host/clonality: rb, polyclonal
supplier: Novus Biologicals
catalognumber: NBP2-15339
lot number: A-3
dilution: 1:1000
Ventana program: CC1st

Antibody: PalmD
host/clonality: rb polyclonal
supplier: Novus Bio
catalognumber: NBP2-55156
lot number: R31253
dilution: 1:750
Ventana program: CC1st

Antibody: Tenascin c
host/clonality: BC-24, ms monoclonal
supplier: Sigma-Aldrich
catalognumber: SAB4200782
lot number: 107M4823V
dilution: 1:1000
Ventana program: CC1m

Antibody: c-myc
host/clonality:rb, monoclonal
supplier: Zeta Corporation
catalognumber: Z2734RL
lot number: ZR02042022A-2
dilution: 1:25
Ventana program: CC2 OptiView

Validation

All antibodies were tested using postive and negative controls. Further information regarding the validation and details of antibody

was as mentioned on the respective company websites :
1. Tenascin c : https://www.sigmaaldrich.com/DE/de/product/sigma/sab4200782
2. PalmD : https://www.novusbio.com/products/palmd-antibody_nbp2-55156#datasheet
3.c-myc: https://zeta-corp.com/product/c-myc-2/
4.ALDH1A3:https://www.novusbio.com/products/aldh1a3-antibody_nbp2-15339

Eukaryotic cell lines

Policy information about cell lines and Sex and Gender in Research

Cell line source(s)	The human Medulloblastoma cell lines DAOY (Ca#HTB-186) and D283med (Ca#HTB-185) were obtained from ATCC, Manassas, VA, USA. UW473 was kindly provided by Michael Bobola. All lines were used as Standards for TMT batches. Cells were cultivated in DMEM (Dulbecco's Modified Eagle Medium, PAN-Biontech) supplemented with 10 % FCS at 37°C, 5 % CO2.
Authentication	Cell lines were authentificated via PCR-single-locus-technology. 16 independent PCR-systems D8S1179, D21S11, D7S820, CSF1PO, D3S1358, TH01, D13S317, D16S539, D2S1338, AMEL, D5S818, FGA, D19S433, vWA, TPOX and D18S51 were investigated. Data was compared with the online database of the DSMZ (http://www.dsmz.de/de/service/services-human-and-animal-cell) and the Cellosaurus database (https://web.expasy.org/cellosaurus).
Mycoplasma contamination	All cell lines were tested negative for Mycoplasma contamination.
Commonly misidentified lines (See ICLAC register)	No commonly misidentified cell lines were used in this study.

Clinical data

Policy information about clinical studies

All manuscripts should comply with the ICMJE guidelines for publication of clinical research and a completed CONSORT checklist must be included with all submissions.

Clinical trial registration	Single patients were enrolled in the SIOP PNET5 study of the HIT-MED study center (for details see Supplementary data file 1c, Supplementary data file 11, survival data for these patients was excluded from further analysis. (ClinicalTrials.gov ID:
Study protocol	NCT02066220) https://clinicaltrials.gov/ct2/show/NCT02066220
Data collection	Data was collected dependent on tissue availability of FFPE Medulloblastoma samples of tumors within the years 1976-2021 were obtained from tissue archives from various neuropathology units in Germany.
Outcomes	Overall survival was assessed via Kaplan Meier Analyses. Survival data from patients enrolled in the SIOP PNET5 study were excluded.
